# Direct participation of people with communication disabilities in research on poverty and disabilities in low and middle income countries: A critical review

**DOI:** 10.1371/journal.pone.0258575

**Published:** 2021-10-14

**Authors:** Caroline Jagoe, Caitlin McDonald, Minerva Rivas, Nora Groce

**Affiliations:** 1 Department of Clinical Speech and Language Studies, Trinity College Dublin, Dublin, Ireland; 2 Institute of Ethics, History, and Humanities, University of Geneva, Geneva, Switzerland; 3 Institute of Epidemiology & Health Care, University College London, London, United Kingdom; University of Washington, UNITED STATES

## Abstract

**Introduction:**

An estimated 1 billion people with disabilities live in low and middle income countries, a population that includes people with communication disabilities (PwCD). PwCD are a heterogenous group with a wide range of abilities who may be underrepresented in research due to the communication demands involved in research participation.

**Methods:**

A critical analysis of 145 studies from a previously published systematic review was undertaken with the aim of documenting the opportunities for direct participation of PwCD in research on poverty and disability in low- and middle- income countries.

**Results:**

The key finding was the high risk of underrepresentation of PwCD in research on poverty and disability in LMICs, despite low rates of explicit exclusion (n = 8; 5.5%). A total of 366 uses of data collection tools were analysed (255 unique tools). The majority of data collection tools had high communication demands (92.9%), including those measuring disability (88.6%) and those assessing poverty (100%). Only 22 studies (15.2%) specifically included PwCD. A subset of these studies (n = 14) presented disaggregated data in a way that allowed for analysis of outcomes for PwCD, suggesting a clear intersection between poverty and communication disability, with findings related to general poverty indicators, reduced access to education, low levels of employment, and additional expenditure.

**Conclusions:**

The findings suggest a systematic underrepresentation of PwCD in research on poverty and disability with substantial implications for future policy and program planning, directly affecting the availability and provision of services and resources for this population. A failure to provide adequate opportunity for participation of PwCD in research risks leaving those with communication disabilities behind in the pursuit of global poverty eradication.

## Introduction

A key recommendation by the World Report on Disability [1], and the United Nations Disability Inclusion Strategy [2], is that more proficient data collection occur in terms of disability prevalence and service needs to ensure that populations facing poverty and marginalisation are provided for in policy and outreach programs, on an equal basis with others. The deficit of sufficient amounts of quantitative and disaggregated data was identified by both reports as negatively impacting on the accurate assessment of the needs of people with disabilities, arguably putting these groups at risk of being ‘left behind’ in the pursuit of global poverty eradication.

A communication disability is defined broadly for the purposes of this paper, adapted from the useful functional definition implied in the Washington Group Questions [3] as: a disability arising from an impairment which limits the ability to effectively and efficiently expressing oneself or understanding others when using a preferred language. Communication disabilities by this definition, include conditions such as aphasia (an impairment in language processing and production commonly follows stroke), dysarthria (a difficulty in speech production which commonly occurs in neurological conditions such as Parkinson’s disease, Motor Neuron Disease), cognitive-communication disorders (affecting many people with dementia or traumatic brain injury), and communication difficulties associated with developmental conditions (such as intellectual disability, developmental language disorder). Hearing difficulties and deafness are *not* included in the definition for the purposes of this paper for two reasons, the first being that hearing difficulties are categorised as distinct in the WGQs and although communication challenges may result these are not inevitable and secondly, in the case of sign language users, not relevant when the individual is using their preferred language. People who are Deaf (individuals with hearing impairments who are members of a Deaf community), deaf (individuals with hearing impairments who are not linked to a Deaf community) or hard of hearing also face difficulties in communicating and therefore, as an established body of research already shows, are frequently excluded from research [e.g. 4, 5]. While these issues overlap, the decision to focus broadly on people with a range of communication difficulties in this paper, as defined by the WGQs, allows for a broader discussion on barriers to inclusion in research specifically faced by this heterogenous population.

Individuals with communication disabilities have a wide range of disabilities: however they may have difficulty understanding others and/or may *sound* different to ‘typical communicators’ affecting how easily they are understood by others. They may use other modalities of communication to supplement verbal communication or as an alternative to verbal communication (such as gesture, writing, communication boards or communication devices). A communication disability may occur in isolation, be associated with other disabilities (such as intellectual disability or cognitive impairments in dementia), or may co-occur with other functional difficulties.

The nature of communication disability impacts on how people are included in research, with true participation necessitating adaptations to consent processes and data collection methods. Reviews in other domains (e.g. stroke research) have suggested that those with communication disabilities are “systematically excluded” [6, pg.193]. The underrepresentation of people with communication disabilities in research significantly impacts on the accuracy of data pertaining to their service [7], and their rehabilitation needs [8]. The existing literature exploring the inclusion of people with communication disabilities in research has largely been conducted in high income countries (HICs) where speech and language therapy services exist. The inclusion of those with communication disabilities in research may be even more challenging in less resourced contexts. Thus, the aim of this study is to critically review existing research on poverty and disability in low- and middle- income countries (LMICs) to examine the extent to which people with disabilities are included in research in general and people with communication disabilities in particular. We are further interested in determining whether appropriate adaptations are being made to data collection tools to support participation in research; and finally, to synthesise specific findings related to poverty and communication disability.

### Communication disability in a global context

Global rates of communication disability are not known. Conservative projections indicate that there will be 190.5 million people with communication disabilities in LMICs by 2025. The existing data is largely geographically and historically framed, but suggests that among people with disabilities, as many as 28–49% have a communications impairment as a component of their disability [9–11]. Reported prevalence is inconsistent and lacks validity for two main reasons: the definitions of disability generally, and communication disability specifically, and the bundling of communication disabilities with other sensory impairments [7]. Even when communication impairments are included in research questions, variability in how the communication disability is defined can negatively impact on the data retrieved. Mulhorn and Threats [12] investigated the prevalence of communication impairment in four HICs, finding that the heterogeneity of definitions used to describe communication impairments lowered the sensitivity of data collection tools. For example, the prevalence of communication impairments in adults, based on the survey question used in the United States, was 0.7%. However, when studies use more robust and inclusive questions that figure increases significantly to 10% of the general population [13]. The second challenge relates to the common practice of ‘bundling’ communication disabilities with data regarding sensory difficulties, and, if a communication disability co-exists with another impairment, disregarding it in favour of the more obvious condition [7]. ‘Multiple disabilities’ is a frequent category used in research and is problematic in terms of identifying populations with specific accessibility needs, such as communication supports. The use of the WGQs has resulted in more specific data on communication disabilities in some contexts although the prevalence figures vary substantially, for example, ranging from 0.4% (e.g. in Vanautu, [14]), to 0.9% (e.g. Guatamala National Disability Study [15]), to 4% (in Syria, [16]). This variation highlights the need to further explore the methods applied in data collection processes to measure communication disabilities.

Participation and inclusion of people with communication disabilities in research. The documented exclusion of PwCD from health research appears to be particularly pervasive for those with moderate to severe communication disabilities (e.g. in aphasia research [17]). This type of exclusion may lead to inadequate service provision and gaps in the evidence base used to inform clinical management of disabled populations [e.g. 6, 18]. Exclusion is difficult to justify given the availability of strong evidence indicating the ability of PwCD to participate fully in research [e.g. 19]. By reducing reliance on rapid spoken interaction and adopting a ‘total communication’ approach, such as increasing the use of images and gesture, PwCD can actively participate in studies. While the use of local languages [20], braille or sign languages interpreters [21] are recognised as necessary for accurate data collection, limited attention has been given to the unique and diverse needs of PwCD.

The inadequate representation of PwCD in research (including research based on census data) may be impacted by the fact that many disability measurement or screening tools do not specifically account for communication disability. This challenge is partly addressed by the adoption of the WGQs. In their short form, the WGQs are a set of six questions aiming to identify people with a disability, with the final question directly addressing communication difficulties. Although the WGQs (when administered as intended) should contribute to improved data disaggregation regarding the prevalence and severity of communication disability, true inclusion of this population is not guaranteed unless the relevant data collection processes (such as interview or surveys) are communicatively accessible.

### Poverty

The extent of poverty experienced by PwCD in LMICs, is unknown, with limited data available. Research in HICs, in which resources and support are more readily available, indicates that those with communication disabilities may be economically disadvantaged. For example, in his analysis of epidemiological and economic data, Ruben [22] concludes that PwCD are disproportionally prevalent in low socio-economic groups, citing data from the US Bureau of Labor Statistics which indicates working-age adults with more severe difficulties in speech had the highest rates of unemployment–up to 75.6%. This rate of unemployment is disproportionately high when compared to that reported for working age people with disabilities overall, which ranges from 50.2% [23], to 64.5% [24] although higher rates of unemployment are reported in ‘developing countries’ [25]. Similarly, working-age men with impairments in speech were 8 times more likely to be unemployed in the United Kingdom when compared to those without disabilities [22]. The effect of a communication impairment on the maintenance of poverty is likely to be more pronounced in LMICs due to socioeconomic factors and lack of access to rehabilitation services which could support the persons return to employment [8]. A lack of access to support and rehabilitation services for PwCD in LMICs is likely to negatively impact on the ability of working age adults to gain or return to employment, maintaining their vulnerability to poverty. For example, Ethiopia has only 1 Speech and Language Therapist (SLT) for every 100 million people [26], in comparison to the UK which has approximately 1 SLT for every 3800 people (based on figures from RCSLT) [27].

Poverty is generally accepted to be multidimensional, encompassing income, the ability to satisfy basic human needs such as shelter, education, employment or healthcare, and the capacity to contribute to family, social and political life [28, 29]. This definition of poverty recognizes not only financial poverty but, also, the marginalisation and exclusion of the poor from society [30]. For the purposes of this paper, the focus is on economic measures of poverty, (income, expenditures, assets and/or socioeconomic status), as these were the dimensions of interest in the previously published systematic review [31], from which the primary studies were identified. There is evidence to suggest that people with disabilities are more vulnerable to poverty, especially in LMICs and that disability is “both a cause and a consequence of poverty” [32, pg.1]. There are several reasons for the bidirectional relationship between poverty and disability. The risk of disability increases with poverty due to the associated lack of access to healthcare, malnutrition, poor living conditions, and inadequate water and sanitation facilities [33]. Conversely, disability can lead to, or exacerbate, poverty. People with disabilities are overrepresented amongst the unemployed when compared to the general population [34], posing a particular challenge for women with disabilities [35, 36]. Educational opportunities may be denied to children with disabilities, impacting their future earning potential [34, 37]. If people with disabilities are prevented from being employed, either implicitly or explicitly, then they may not be able to earn a living. Additionally, upon gaining employment people with disabilities earn less than those who do not have a disability, even though they are conducting the same work [38]. The stigma often associated with disability contributes to the barriers faced and the experience of disability, resulting in further marginalisation [39, 40].

An understanding of what disaggregated findings already exist and to what extent they are representative of PwCD and based on inclusive research practices, is an important first step in ensuring an inclusive evidence base which informs the specific needs of PwCD.

## Methods

The aim of this critical review is to document the opportunities for, and actual participation of, PwCD in research on poverty and disability in LMICs that met the criteria for inclusion in a previously published systematic review [31]. Specifically, this study aimed to investigate:

1. The extent to which data collection tools and processes reported by primary studies addressing poverty and disability allowed for the direct participation of PwCD (including sampling / recruitment processes, the communication demands of data collection tools and the extent of any adaptations to data collection tools reported)
2. Where disaggregated data is available, to synthesise the findings on poverty and PwCD in LMICs

### Research design

A critical review approach was adopted, allowing for extension beyond description in order to analyse the primary studies in depth. A critical review approach differs from a systematic review in that it is typically not based on a structured systematic search of the literature, but is focused on a conceptual evaluative approach to the available literature [41]. The focus of this critical review was specifically on the details of the methods reported, particularly focused on the inclusion and exclusion criteria, the data collection tools and processes, and any aggregated findings reported for PwCD. This critical review is approached through analysis of papers identified by a previously published systematic review that has received high levels of attention (citation count of 162 in April 2021, Google Scholar).

### Sources of data

The papers included in the previously published systematic review, *Poverty and Disability in Low- and Middle- Income Countries* [31] were eligible for inclusion in the critical review reported here. The authors of the systematic review [31] searched ten electronic databases in 2016 (EMBASE, PubMed, MEDLINE, Global Health, Web of Knowledge, Academic Search Complete, EconLit., ERIC, Social Policy & Practice, and FRANCIS) for studies, published between 1990 and March 2016, which examined the relationship between poverty and disability. The published systematic review followed stringent protocols in line with those recommended for systematic reviews (PRISMA for the published study is available in the open access publication, [31 p.9]). Of the 15,500 articles generated during the initial database search, 150 studies were found to meet their criteria. These 150 studies were then considered as eligible for inclusion in the critical review reported here.

#### Missing data

One study was untraceable during the course of this critical review: Febrile illness and pro-inflammatory cytokines in the first year of life predict impaired child development in Bangladeshi infants living in poverty [42]. However, Febrile illness and pro-inflammatory cytokines are associated with lower neurodevelopmental scores in Bangladeshi infants living in poverty [43] appears to be a version of the original article as it was produced by the same authors and has a similar title, and thus was used in place of the untraceable study. Additionally, the full text of four studies (Table 1) were inaccessible to the researchers. The final exclusion related to a published abstract which did not include sufficient information for analysis. As a result, 145 studies were used as the data set for this critical review (see S1 File).

**Table 1:** Papers excluded from critical review.

Study	Reason for exclusion
The Nakuru posterior segment eye disease study: methods and prevalence of blindness and visual impairment in Nakuru, Kenya [44]	Full text not available at time of review
Prevalence and distribution of cognitive impairment in dementia (CIND) among the aged population and the analysis of socio-demographic characteristics: the community-based cross-sectional study [45]	Full text not available at time of review
Poverty and musculoskeletal impairment in Rwanda [46]	Full text not available at time of review
Prevalence and risk factors of cognitive deficits and dementia in relation to socioeconomic class in an elderly population of India [47]	Full text not available at time of review
Factors associated with cognitive impairment in older adults: A population based survey, South Brazil [48].	Published abstract–insufficient data for analysis

### Data extraction and analysis

**Fig 1:**
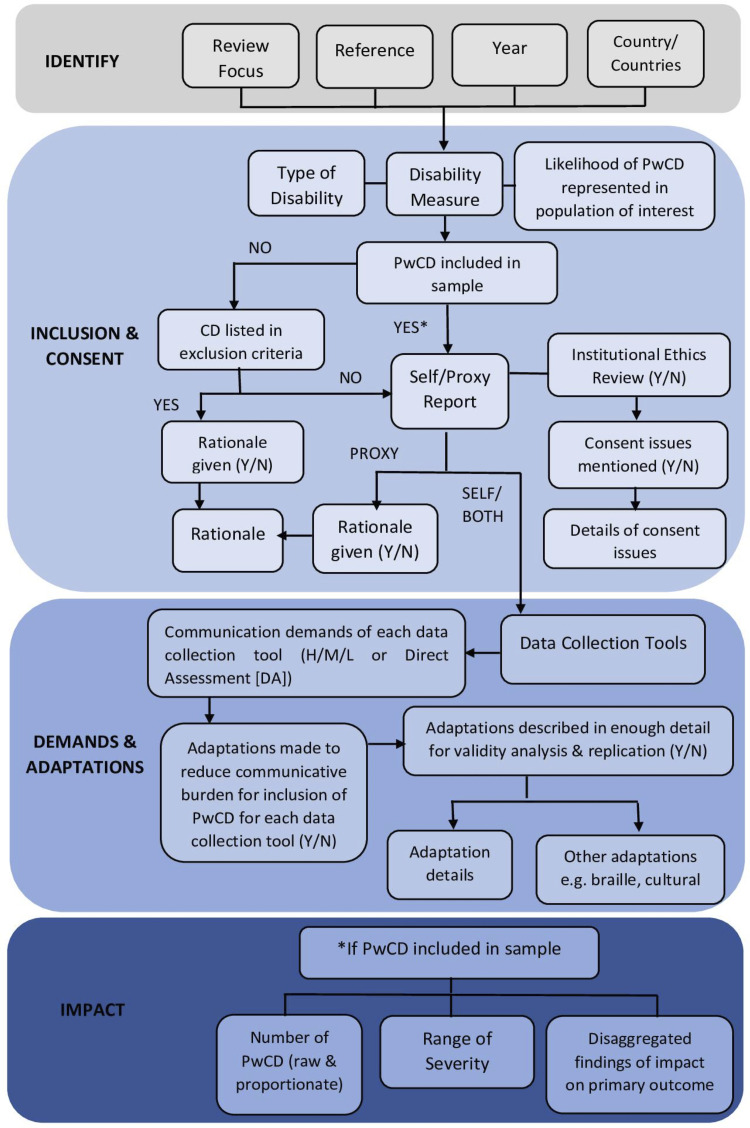
Data extraction flowchart.

A data extraction form, which was pre-piloted and standardised, was used to collate information drawn from each of the 145 studies. The data extraction process is illustrated in Fig 1 and corresponds to the 4 sections of the data extraction sheet developed in Excel. Citation details (including the country/countries involved) were captured in section 1; section 2 captured details relevant to research question 1: inclusion criteria and consent, including any reference to communication considerations and the likelihood of PwCD being represented in the sample; demands and adaptations, including an evaluation of each data collection tool reported in the study and details of any adaptations reported; finally, section 3 captured the reported findings relevant to any disaggregated data on PwCD (addressing research question 2). Any data which reported on poverty-related findings for PwCD, were then explored using a quantitative content analysis. The extracted data on poverty and communication disability was read and re-read to identify categories of meaning, which were then quantified through frequency counts. In order to ensure reliability, Prior to the initiation of data extraction, the first 10 studies were jointly reviewed between two researchers (C.J. and C.McD.) in order to identify, discuss and resolve any discrepancies. Subsequently, a random numbers generator was used to select 15 studies from the remaining 135 papers and the reviewers independently extracted data from these. Cohen’s Kappa Statistic (K) was used to calculate inter-rater agreement. Agreement ranged from K = 0.54 (moderate agreement) to K = 1.0 (complete agreement) with an average level of 0.78 (substantial agreement) [49]. As a result of the agreement scores, one reviewer (C.McD.) extracted data from the remaining studies.

### Consent considerations

Over the years, ethical practices on research involving human participants has developed substantially. Today Institutional Research Boards are well established institution and their role is to ensure that that research is conducted in an ethical manner according to relevant legislation [50]. One aspect of ethical research is a clear and transparent consent process, considered to protect the rights of research participants and minimising coercion [51]. For traditional notion of informed consent, competence is the ability to comprehend information and make an autonomous decision [52]. Cognitive functional and communication diversity has not always being properly dealt with and have often been use to exclude and discriminate person with disabilities [53]. Thus, It is possible for PwCD to give consent to their participation in research when the appropriate adaptations are made to consent processes and sufficient support is available to them [e.g. 6] and these adaptations should be reported to the IRB or in the paper. Over scrutiny on consent practices for persons with disabilities have at time overlook broader issues on fairness, dignity and justice [54, 55]. Due to the perceived challenge of gaining consent from PwCD [6], consent issues reported by studies were recorded to determine if challenges specific to communication disability were being encountered by researchers.

### Defining inclusion and exclusion of PwCD

Data was extracted as to whether PwCD were explicitly excluded (any type of communication impairment was listed in the exclusion criteria); included (the sample reported on people with communication disability); or potentially included (the research design and the sample strategy indicated that PwCD could have formed part of the sample) (Table 2).

**Table 2:** Definitions of inclusion and exclusion of PwCD in primary studies.

Description	Definition
**Explicitly Excluded**	Refers to cases in which PwCD are explicitly excluded by virtue of the exclusion criteria of the study
**Potentially Included**	Refers to cases in which there is no explicit mention of PwCD in the study or the findings but given the sample population and research question, there was the opportunity for PwCD to participate in the research
**Included**	Refers to cases in which PwCD are explicitly mentioned in the study and/or when data about PwCD is reported (e.g. raw or proportionate numbers of PwCD in sample)

In recognition of the fact that some selected study populations may have a higher likelihood of including PwCD than others (e.g. research participants with neurological conditions where communication disability is common verse participants with visual impairments), a likelihood scale was devised for the purposes of this study (Table 3).

**Table 3:** Likelihood scale.

Likelihood	Examples	Rating
**Unlikely**	Communication disability is listed as an exclusion criterion	1
**General population**	Censuses & studies on people with disorders not directly associated with CD (e.g. visual impairment).	2
**Higher than general population**	Psychiatric disorders (schizophrenia) & older adults (over 60), where communication difficulties have been documented in the literature	3
**Highly Likely in study population**	Dementia, Alzheimer’s Disease, cognitive impairment, intellectual disability & ASD or other disorders where communication difficulties form part of the presentation of the disorder	4

Using a scale from 1 to 4, likelihood was assigned on the basis of the article’s research question and its sampling strategy as these are not mutually exclusive. For example, based on the research question alone, a study investigating the relationship between food security and visual impairment would be assigned a likelihood number of 2 (PwCD should be represented in this sample at a similar rate to their representation in the general population of people without a disability). However, if when the sampling strategy is examined, it is found that the researchers specifically assessed those over the age of sixty, the chance that PwCD would be present in the population increases given that the risk of having a communication disability increases with age [56].

### Defining communication demands

Each study that either explicitly or potentially included PwCD were further analysed in relation to each of the data collection tools used, both in terms of measurement of disability and in terms of measures of poverty. Each tool was analysed independently. The communication demands of the data collection tools and processes were analysed according to four categories in a system designed for the purposes of this study: high communication demands (defined as complete reliance on questions/probes which were provided in verbal format only and requiring responses in verbal format); medium communication demands (in which questions/probes were provided in verbal or written format and visual support, such as a visual analogue scale, was available to support responses); low communication demands (in which visual supports were available during questioning, or adapted for individual participant needs and any modality of response was accepted and anticipated); and direct assessment (in which the data collected was not reliant on communicative responses). Full descriptions of these categories are outlined in Table 4. These categories were applied to all measures identified in the studies, encompassing assessments of poverty, assessment of disability, and tools which incorporated both poverty and disability measures. Given that data collection processes can be adapted to make them more accessible, details were extracted related to any reported adaptations that had the purpose of reducing the communicative demand and facilitating the inclusion of PwCD in the research.

**Table 4:** Definitions of communication demands of data collection tools.

Category and definition	Possible Examples
**High Communication Demand:**	Structured interview with no visual supports.
Questions/probes provided in verbal format or written format only and responses expected in same format.	
	Written census with no additional supports.
	Semi-structured interview with no visual supports.
**Medium Communication Demand:**	Interview with the support of a visual analogue scale.
Questions/probes provided in verbal/written format and visual support available during responses.	
**Low Communication Demand:**	Interview with adaptations for PwCD e.g. visual response modes, interpreters and note-taking for gestural responses.
Visual supports were available during questioning, or adapted for individual participant needs and any modality of response was accepted and anticipated.	
**Direct Assessment:**	Physical examination (e.g. weight or height assessment to evaluate nutritional status; optometry evaluation; hearing assessment).
The assessment is carried out directly on the client and they do not need to communicate with the assessor in order for the examination to take place.	

### Identifying primary outcomes for PwCD

If PwCD were included in studies then data relating to the outcomes of PwCD in those specific studies was recorded. The raw and proportionate number of PwCD included in studies was documented. The degree of severity of communication disability encountered in the studies was also recorded, if reported explicitly by the authors of the primary studies and any data on the poverty-related outcomes for PwCD were synthesised using content analysis.

## Findings

The findings are presented in two parts. Firstly, the potential for direct participation of PwCD in the data collection processes is presented. Secondly, a synthesis of findings on the interaction between poverty and communication disability is presented, based on the subset of studies (n = 22) in which PwCD were included.

### The potential for direct participation of PwCD

The potential for direct participation of PwCD was explored through analysis of the consent considerations reported in the studies (that is, whether concerns about communication difficulties impacting on the ability to give consent were a factor in exclusion); the reported inclusion and exclusion of PwCD; and the communication demands of the data collection tools and processes. An overview of the findings is presented in Table 5.

**Table 5:** Overview of findings across studies (n = 145) categorised by potential for inclusion of PwCD.

	Number (%) of studies	Number (%) reporting consent considerations	Number (%) with likelihood rating > 2*	Number (%) in which all data collection tools had high communication demands
**Explicitly excluded**	8 (5.5%)	0	3 (38.5%)	n/a
**Potentially included**	115 (79.3%)	13 (11.3%)	60 (52.2%)	110 (95.7%)
**Included**	22 (15.2%)	2 (9.1%)	2 (9.1%)	20 (90.9%)

*Likelihood rating of >2 indicates that the likelihood of the study sample including PwCD is higher than that of the general population.

#### Consent considerations

Consent considerations were reported in 10% (n = 15) of studies. A participant’s ‘lack of capacity to consent’ was the most frequently occurring concept, reported by 7 studies. This was followed by literacy difficulties impacting on the consent process in 6 studies. There were three additional consent consideration concepts outlined by studies; intellectual disability or mental health (n = 2), stigma (n = 1) and cultural differences (n = 1). No specific mention was made suggesting that communication difficulties were considered to impact directly on capacity or consent.

#### The inclusion and exclusion of PwCD in studies on disability and poverty

Eight studies (5.5%) explicitly excluded PwCD from their research. Of these, two studies [46, 47] provided a rationale for this exclusion criterion. A rationale for exclusion was considered to have been provided if the communication disability was linked to the research question or to the data collection methods. In both cases, the rationale for exclusion linked the communication difficulties to an inability to participate in the data collection process, for example citing the exclusion of those who had communication difficulties as due to their inability to respond to questions “personally” [57, pg.3]. The remaining seven studies cited communication difficulties as the reason for the exclusion of PwCD from research, with no further rationale (Table 6).

**Table 6:** Rationale for explicitly excluding PwCD from research.

Study (Disability Focus)	Description of exclusion criteria provided by study authors *(italics added)*
Fernández-Nino, et al. 2014 [57] (Older adults & depressive symptoms)	“Of the 8,874 OA [Older Adults] who participated in ENSANUT [National Health & Nutrition Survey, Spanish initials] 2012, we excluded those who were *unable to respond to the interview personally*. The principal reasons for failure to respond were hearing or speech impairment (694 OA) and memory loss (320 OA).”
Guo et al. 2015 [58] (Depression)	“The criteria for inclusion were those who had experienced the earthquake and could understand all questions on the study protocol or *communicate with interviewers*.
Arguvanli et al. 2015 [59] (Cognitive Impairment in older adults)	“Individuals who had severe deficiency of hearing or eyesight, *had severe problems with communication*, or were unable to reach an ASM [Turkish abbreviation for a Family Health Centre] were not included in the sample.”
Awas et al. 1998 [60] (Mental Health Disorders)	“Exclusion criteria for respondents were refusal to participate and *incoherent speech* because of severe illness or old age.”
Cockburn et al. 2012 [61] (Visual impairment)	“Out of 3100 eligible people, 2750 (89%) were examined, 169 (5.5%) were not available, 170 (5.5%) refused and *11 (0*.*4%) were unable to communicate*.” Participants who could not communicate were then excluded from the study.
Gawde et al. 2013 [62] (Mental Health Disorders)	“Twenty-nine of them refused to participate in the study, 89 were not available at house even after four visits, 18 could not be interviewed due to language barriers or *communication problems* whereas another 22 left the interviews half way.”
52. Kuper et al. 2008 [63] (Visual Impairment)	“Case and control participants who were significantly *communication impaired* (e.g. deafness, dementia, or psychiatric disease) were excluded (fewer than five per country), and one case was excluded in the Philippines because of missing age data.”
Sengupta et al. 2014 [64] (Cognitive Impairment associated with Ageing)	“Those who were blind, or hearing and/or *speech impaired*; those with diagnosed psychiatric illness (schizophrenia, mental retardation); and those who were too ill at the time of the study, were also excluded, as it would have been difficult to obtain reliable information from them.”

Likelihood ratings described in Table 4: 2 –rate of communication disability expected at the level of the general population; 3 –rate of communication disability estimated to be at a higher rate than general population; 4 –communication disability highly likely in the study sample

The majority of studies (n = 115) did not list communication disability as an exclusion criteria, nor was this group identified in the sample, meaning that PwCD were potentially included in 79.3% of the studies. Of these 115 studies, 110 (95.7%) relied exclusively on data collection tools that were characterised by high communication demands. The remaining papers used either direct assessment, in which the data collected was not reliant on communicative responses (e.g. meaures associated with estimating nurtitional status, such as weight or height) (n = 4; 3.48%) or prevalence estimates based on secondary analysis of epidemiological data (n = 1). Fifty-five studies addressed populations in which communication disability would be expected to be at a similar level to that of the general population of people without disabilities. Forty-seven of the studies which potentially included PwCD addressed poverty in populations in which the likelihood of communication disability was above that of the general population. Older adults, for example, have a higher prevalence of communication disability [56] and communication impairments are associated with schizophrenia [see 65]. Thirteen studies were conducted within populations in which there was a high likelihood of communication disability. The likelihood of passive exclusion in studies which potentially included PwCD, was therefore considered to be notable in at least 60 studies (52.17% of the studies which potentially included PwCD), particularly given that all 60 utilised data collection tools with high communication demands.

Of the 145 studies, 22 (15.2%) reported on PwCD in their findings, meaning that their sample clearly included PwCD, based on the details or findings reported. Seven of these studies addressed disability in children, 4 in adults, and the remainder (n = 11) addressed both children and adults. Over half of the studies (n = 14) reported on poverty indicators in a way which data specific to PwCD could be extracted. Twelve studies specified the raw or proportionate number of PwCD in the sample; 31.8% (n = 7) used the WGQs, and 1 used the Washington Group Child Functioning Set. The percentage of PwCD across the 12 studies reporting this data, ranged from 0.09% (n = 2,510 of a total study sample of n = 2,526,145) to 62.90% (n = 760 of a total study sample of n = 1,208). The mean percentage of PwCD included in each study was 10.87% and the median percentage of PwCD included in each study was 3.6%. The total number of PwCD included in the 12 studies was 11,910. The proportionate percentage equated to 0.43% (n = 11,910 of a combined study sample of n = 2,751,516) (Table 7). The majority of studies that included PwCD (n = 20) were conducted in study populations in which the prevalence of communication disability was considered to be at the level of the general population, although the rates of communication disability ranged from 0.09% to 36.86%. One study [66] was conducted in older adults and considered to have a higher likelihood of communication disability being represented, although the percentage of PwCD in the study was only 4.19%. The final study of this subset [67] addressed dementia associated disability and was therefore considered to have a high likelihood of representation of PwCD, with the percentage of PwCD being the highest across all studies at 62.90% of the sample.

**Table 7:** Raw and proportionate number of PwCD in studies reporting disaggregated data.

Study	Rating*	Raw number of PwCD in study	Total number of study participants	Percentage of PwCD in each study	Tool used for report of Comm. Disability
Jiang et al. 2014 [43]	2	72	398	18.09%	WGQ-SS
Wandera et al. 2014 [66]	3	110	2,628	4.19%	WGQ-SS
Li et al. 2015 [67]	4	760	1,208	62.91%	China National Survey on Disability
Wang et al. 2015 [68]	2	2,510	2,526,145	0.10%	China National Survey on Disability
Pham et al. 2013 [69]	2	93	9,882	0.94%	WGQ-SS
Danquah et al. 2015 [70]	2	38	3,132	1.21%	WGQ-SS
Subbaraman et al. 2014 [71]	2	4	209	1.91%	WHODAS 2.0
Li et al. 2015 [72]	2	5,048	161,478	3.13%	China National Survey on Disability & WHODAS 2.0
Natale et al. 1992 [73]	2	28	640	4.38%	WHO 10 Question Screen (TQS)
Marella et al. 2015 [74]	2	11	195	5.64%	Rapid Assessment of Disability
Kuper et al. 2014 [75]	2	2,241	8,900	25.18%	Binary disability question, follow-up question on type & duration
Mont and Cuong 2011 [76]	2	995	36,701	2.71%	WGQ-SS
**Total (proportionate percentage)**		**11,910 (0.43%)**	**2,751,516**	-	
**Mean**		**993**	**229,293**	**10.87%**	

*Likelihood ratings described in Table 3: 2 –rate of communication disability expected at the level of the general population; 3 –rate of communication disability estimated to be at a higher rate than general population; 4 –communication disability highly likely in the study sample

Note: in some cases the studies reported total participants and percentage of PwCD, in which case the raw number was calculated.

Data regarding additional or co-occurring disabilities was not available. In studies which reported on multiple disabilities, these were grouped without specification as to the nature of the other disabilities. The degree of communication disability (or severity) for PwCD, was infrequently reported in studies, with only six studies reporting on ‘severity’. Those studies which included a measure of severity used either the WGQs or were data drawn from the Chinese National Sample Survey on Disability. The tools used and response options were diverse, including “disabled or extremely disabled,” [77] and “mild or serious disability” [78] and when severity was included in the data collection tool, disaggregated numbers were not always reported. The heterogeneity of severity descriptors used, even with only two disability measures, made analysis across studies difficult.

### Communication demands of data collection tools

**Fig 2:**
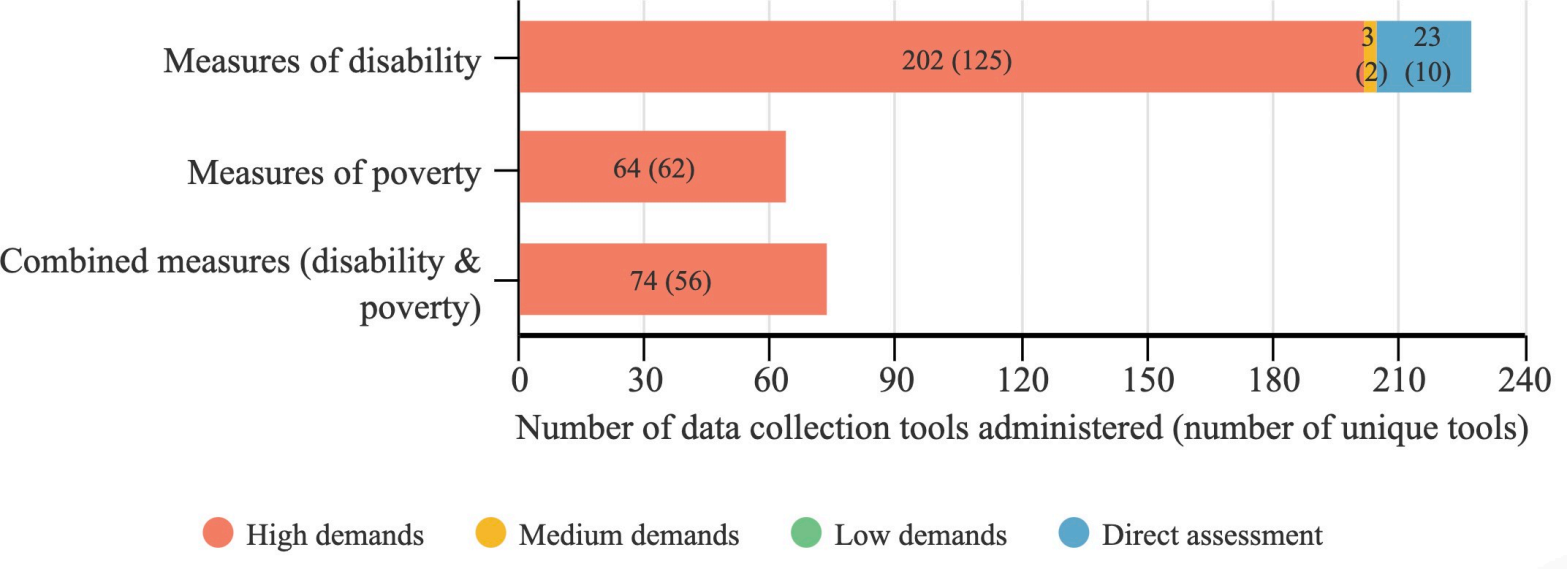
Number of data collection tools by communication demand.

In total, there were 366 data collection tools used, with 255 unique tools used, across the 145 studies included in this critical review. Each use of a tool was counted and analysed individually to ensure that any adaptations across studies could be captured. The tools analysed included any tools used to collect data on disability (n = 228 tools, n = 137 unique tools); tools addressing income, livelihoods or other dimensions relevant to poverty (n = 64, n = 62 unique tools); and tools with a dual purpose to explore both poverty and disability, such as interviews or household surveys comprising both elements (n = 74, n = 56 unique tools). The analysis was based on the reported characteristics of the tool and its administration available in the study, or in relevant protocol papers referenced by the study authors in each case. Fig 2 presents the data collection tools classified by communication demand.

Of the tools which addressed disability, the majority (n = 202; 88.6%) had high communication demands. Three tools (1.3%) had medium communication demands, with two representing the same tool, the Sheehan Disability Scale (Sheehan, 1983), deployed as designed across two studies (P47, P123). Direct assessment was used by 23 tools (10.1%). Of the tools designed to assess poverty, usually used at the household level, all (n = 64, 100%) had high communication demands. Combined tools, in which poverty and disability were measured, were all classified as having high communication demands (n = 74, 100%).

There was no data collection tool categorized as having low communication demands. There were no reported adaptations made to tools with medium communication demands, rather the lower demand was a function of the original design of the tool (such as the use of a visual analogue scale in the study by Sharifi and colleagues [79]). No adaptations to data collections tools were reported in relation to accommodating people with other disabilities, such as braille versions of questionnaires, or the use of sign language interpreters, although translation of tools and/or cultural adaptation, was reported in 32 of the 145 studies included.

Within studies which reported findings for PwCD, 42 data collection tools were used, 13 of these being disability measurement instruments. Although these studies explicitly included PwCD, the majority of data collection tools, regardless of the study population and likelihood of communication disability, had a high communication demand (n = 40; 95.2% of uses), with the remaining two tools utilising direct observation (e.g. physical examination). No study reported adaptations to their data collection tools to facilitate the inclusion of PwCD in research. These figures suggest that participation in the research may have been particularly limited for those with more significant communication disability due to the inaccessibility of high communication demand tools for many PwCD. However, most studies did not report on the severity of communication disability included in their research, limiting the potential for further analysis.

### Poverty and communication disability: Synthesis of reported associations

Of the 22 studies which explicitly included PwCD in research, 14 (63.6%) studies reported data pertaining to PwCD and eight (36.6%) did not disaggregate data or report on details specific to PwCD to allow for findings specific to PwCD to be extracted. Six categories were identified illustrating aspects of the bidirectional relationship between communication disability and poverty (or proxy indicators of poverty), with varying frequency of occurrence in this subset of studies (Table 8): poorer general poverty indicators (8), education (3), additional expenditure (3), employment (2), and rural/urban divide (2). Critically, many of these indicators may have been affected by co-occurring difficulties (e.g. intellectual disability), and communication disability may have been a complicating factor. However data on the nature of the communication disabilities or co-occurrence with other disabilities was not available to allow for this determination to be made, and thus the synthesised findings likely represent a group with variability in communication needs.

**Table 8:** Categories of findings pertaining to poverty and communication disability.

Category (number of studies coded to category)	Examples of findings from studies with disaggregated data on PwCD
**Poorer General Poverty Indicators (n = 8)**	Households which had a child with communication disability had a significantly lower average income than those households without communication disability [69]
	Data indicated that 6.8/310 children in the lowest social class had a communication disability compared to the-next-lowest social class with 4.2/330 children [73]
	Data indicated that PwCD were more prevalent amongst the poorest cohort (4.12%) as opposed to the richest (1.99%) [76]
	Increased risk of delayed language development in children of mothers who were depressed and living in a context with low family income [80]
**Lower Education / Access (n = 3)**	Communication disability was more common in households where the family head had a lower average education attainment level i.e. no education, primary school graduate and lower secondary school graduate [69]
	Children with learning or communication disabilities were among the least likely to attend school [81]
**Lower Employment rates (n = 2)**	Findings indicated that communication disability was more common in households where the workforce was unskilled [69]
	Data extracted from results tables indicated that PWCD who had mild (77.6%) / severe (64%) impairment were also less likely to be employed compared to typical population (79.8%) [78]
**Additional Expenditure (n = 3)**	Findings indicated that 11.9% of a family’s income was spent on additional healthcare costs when their household included a child with a communication disability (p < 0.001) [69]
	Communication disabilities had the highest additional cost of living with disability amounting to between 24.2% and 32.8% of the household’s income, resulting in lower quality of life [82]
**Rural /Urban Divide (n = 2)**	Tabled data indicated that communication disability was most common in …. rural households (p < 0.044) [69]
	Tabled data indicates that the prevalence of communication difficulties (0.8%) in the sampled area (non-notified slum) was higher than in urban areas (0.14/0.2) sampled during census and national household survey [71]

The most frequent category identified was that of ‘poorer general poverty indicators.’ PwCD were found to have reduced income compared to those who have other disabilities or no disabilities [e.g. 69]. Additionally, children who were born into households earning a low wage were more likely to have a communication difficulty compared to those who belonged to a higher income family [80]. Communication difficulties were also more common among those with a lower socio-economic status [73, 76]. Communication disability or language delay were more common in households with lower educational attainment [59, 70]. Having a communication disability was also found to impact on the school attendance of children, with this group “consistently among the least likely to attend school, particularly in Africa” [75, pg.4]. In terms of employment, PwCD had lower employment rates than the general population even when the impairment was described as ‘mild’ [78] and those with communication disability were more likely to be in a household engaged in less skilled work [69]. Additional expenditure was reported in disaggregated findings for PwCD in 3 studies and while 2 studies reported increased expenditure in families of PwCD, related to either cost of living [82] or healthcare expenditure [69], one study reported low spending due to a lack of treatment options for those with needing SLT [77]. Two studies reported geographical differences in the prevalence of communication disability, with higher rates in rural areas [69] or ‘informal slums’ [71].

## Discussion

In 2015, the United Nations General Assembly (UNGA) published the *Sustainable Development Goals* which state that no one will be left behind in the pursuit of global poverty eradication by 2030 [83]. The findings of this critical review indicate that PwCD lack representation in data on disability and poverty. While disaggregated data does not guarantee improved social or economic response to people in need, it does provide the *opportunity* to identify difficulties and the *potential* respond specifically to needs. The lack of relevant data, arguably amounting to systematic underrepresentation in research on poverty and disability in LMICs, is therefore of particular concern to PwCD and their families.

A key finding of this review was the high risk of underrepresentation of PwCD in research on poverty and disability in LMICs, despite low rates of explicit exclusion (n = 8; 5.5%). Clearly it is legitimate for research articles to have inclusion and exclusion criteria specific to their research question, protecting the accuracy of data [84] and thus the findings should not be interpreted as indicating that PwCD should be included in every study. Instead, our analysis suggests that a more nuanced understanding of inclusion of PwCD in disability-focused research is warranted, beyond a dichotomous categorisation of included/excluded.

Legitimate exclusion criteria (considered for these purposes to be driven by the research question, rather than the practicalities of inclusion) provide transparency and allow for adequate interpretation of the findings. However, PwCD were infrequently identified in the exclusion criteria, meaning that in most of the studies reviewed, individuals with identified communication difficulties were *potentially included* (n = 115; 79%) although the findings were not reported in a disaggregated manner to allow for this determination to be made. In this large subset of studies, PwCD had the potential to be represented in the research, however structural factors and resources are likely to have undermined their direct participation, through passive exclusion. In these cases structural factors (i.e. assumptions of lack of capacity, the privileging of some modalities of communication over others), resources (the lack of communication accessible tools; possibly time pressures for data collection; lack of enumerators with skills in supporting communication), and potentially features of the communication difficulty itself (more severe communication disabilities associated cognitive difficulties), may have impacted on participation. Even in studies in which PwCD were explicitly represented in the sample (n = 22; 15.2% of papers), the high communication demands may have impeded their full participation and resulted in the passive exclusion of those with more severe difficulties.

Without data on the severity of communication disabilities (reported by only 4 of the studies), no clear determination can be made in terms of the relationship between the nature or degree of communication difficulties and participation in research. Lack of detail in reporting what communication supports were used in research on patient satisfaction was similarly found in the review by O’Halloran and colleagues [18]. Given the communication demands of the tools, it seems a reasonable assumption that where PwCD were included in the research they probably had mild to moderate impairments, likely relying on auditory-verbal communication. This hypothesised disproportionate impact in terms of research participation highlights the need for accessible tools to ensure more equal opportunities for representation.

### Data collection tools and processes as resources

Data collection tools are resources, and can either facilitate participation or contribute to exclusion from data collection processes and, as a consequence, from the datasets that result. The majority of data collection tools used to measure disability had high communication demands (88.6%). Tools measuring poverty (whether stand-alone or combined with inclusive measures of disability) all had high communication demands (100%), but as they would typically be administered at household level, direct participation of PwCD would not be required in many cases. Of the 22 studies which included PwCD, 7 focused on children with communication disability. The high communication demands in these instances may be off-set by the use of a proxy, but not in all cases. A parent may themselves have a communication disability, or, in some cases the tool is self-report, relying on auditory-verbal abilities or reading (e.g. Children’s Depression Inventory, Kovacs, 1992), which may be impaired in children with developmental communication disorder, for example. No study reported making adaptations to data collection processes to facilitate the inclusion of PwCD, or indeed of people with disability in general. In forgoing adaptations to data collection tools and routinely using high communication demand tools, researchers are passively excluding PwCD from research, even when the sample appears to include them. This finding builds on existing evidence from O’Halloran and colleagues [18], who outlined how ‘communicatively vulnerable patients’ are at risk of explicit exclusion from research, and by highlighting how this population are also at greater risk of *passive exclusion* from studies.

Palmer and Paterson [19] argue that the full participation of PwCD can be facilitated with the appropriate adaptation of data collection tools, making the extent of this underrepresentation difficult to justify. By introducing communication support strategies, such as images and gesture, and reducing reliance on rapid spoken interaction, researchers can enable PwCD to participate fully in research. These adaptations can also be used to gain consent directly from participants with communication impairments as far as possible (as opposed to their next of kin), and respects the autonomy and decision-making ability of this population. Simple adjustments in tools and processes may increase research participation and hence the representativeness of the sample.

Another striking finding was that 42 *different* data collection instruments were identified in publications reporting on data collected from PwCD (with 13 distinct instruments addressing disability measurement). This large number of data collection tools used by the studies limits the comparability of data and therefore the opportunities to identify and respond to patterns across contexts. There is a need for a commonly used data collection instrument on disability for use in research addressing poverty for example. The lack of a common data collection tool represents a lost opportunity to generate comparable datasets increasing the potential for meaningful analyses. The WGQs have been highlighted as an appropriate tool to generate comparable data that allows for assessment of participation in key activities, such as education and employment. However, in their standard form, the WGQs themselves have high communication demands. We suggest that whatever tools are used to collect data from samples in which PwCD can be expected, are designed or adapted, ideally using co-design processes, in such a way as to support participation such as allowing opportunities for non-verbal response modalities.

### Questions of prevalence and representation

When PwCD were included in the sample in a way which allowed for disaggregation of findings, the findings reflected the wide range of prevalence which has been emphasised by others [e.g. 7]. Only 12 of the 22 studies which explicitly included PwCD documented quantitative data about this population, with the percentage of PwCD in each study ranging from 0.09% to 62.90%. This wide variation appears to be due to the diversity of disability definitions used by studies, the extent of which is evidenced by the heterogeneous classifications of severity used by the six studies that documented communication disability severity. Even when studies had the same authors, communication disability severity classifications were sometimes substantially different. Such diversity lowers the sensitivity [12] and accuracy of data collection tools [7], affecting the validity of subsequent communication disability prevalence figures and limiting the comparability of studies. The lack of reliable prevalence figures is not unique to communication disability, and challenges in collection of data on disability is well documented [see 85]. However, given that data collection often relies on self-report or other communicative engagement with enumerators, PwCD may be at particular risk of being under-represented in disability data. Lack of valid and reliable data has substantial implications for future policy and program planning, directly affecting the availability and provision of services and resources for this population. These services include poverty alleviation initiatives spearheaded in response to Sustainable Development Goal 1, and international development programmes which must be inclusive of PwCD to fulfil the human rights requirements of Article 32 of the Convention of the Rights of Persons with Disabilities.

### Poverty and communication disability

The findings from studies which disaggregated data in a way that allowed for analysis of outcomes for PwCD (14 papers), suggest a clear intersection between poverty and communication disability. However, given that PwCD have a wide range of abilities, communication disability may be a complicating factor, compounding other difficulties or disabilities. For example, PwCD include a number or people who also have intellectual disabilities and their inability to acquire an education and find employment may reflect limitations linked to the combined impact of both disabilities. The lack of clearly disaggregated data with this degree of detail limits the conclusions which can be drawn.

In line with data from HICs [22], PwCD were most prevalent amongst those with a lower socio-economic status [69, 73, 76, 86], and were less likely to be employed when compared to the general population, even when the communication impairment was ‘mild’ [78]. PwCD also had reduced income compared to those with other disabilities or no disability [69, 78, 80]. Although this data offers some insight into the human impact of poverty and disability for PwCD in LMICs, it is likely not representative of all PwCD, particularly given the communication demands of the data collection processes. The synthesis of the outcomes for PwCD are in contrast with findings suggesting that communication disability does not increase the odds of being multidimensionally poor [86]. We suggest that high communication demands inherent in data collection processes may skew findings by inclusion of only those individuals with milder impairments. For accurate determinations to be made on the representativeness of a given sample including PwCD, there needs to be clear reporting of how data collection processes were administered and adapted.

### Towards meaningful representation of PwCD in research

In the context of this paper, we have assumed that most people value the opportunity to be heard and to participate in conversations which may inform services relevant to their own life and wellbeing. Although it is acknowledged that research participation may not be an activity valued by all, it remains critical that the *opportunity* to fully participate exists for all who wish to be represented. One potentially useful way to frame the issues of inclusive data collection and the use of research (or census) data use for social change, is through a Human Rights-Based Approach to Data (HRBAD). The United Nations laid out a set of preliminary principles underlying a HRBAD–participation, data disaggregation, self-identification, transparency, privacy and accountability [87]. Of the seven principles those of participation, data disaggregation and accountability are particularly pertinent to the findings of this study.

In terms of participation, a HRBAD recognises that ‘[a]ll data collection exercises should include means for free, active and meaningful participation of relevant stakeholders, in particular the most marginalized population groups’ [87, p.3]. Passive exclusion of PwCD through the use of data collection tools with high communication demands does not meet this standard of good practice. The UN guidance note does make provision for cases in which direct participation may not be possible, suggesting that these group may include those who are extremely marginalized and lack ‘access, ability or resources to engage productively in participatory processes’ [87, pg.4]. We suggest however that exclusion from direct participation should not be as a result of inaccessible research methods or data collection processes.

The second principle recognises that data disaggregation is required in order to identify patterns of inequalities. The findings of this study suggest that communication disability may be a contributing factor to inequalities and therefore disability data should allow for identification of PwCD. The Washington Group Short Question Set on Disability [3], when administered as intended, would appear to be a useful tool. The increase in inclusion of PwCD starting around 2011, although not statistically significant, may reflect the increased use of the WGQs as it grew from a new methodology being developed by a UN Statistics City group to an established methodology. The WGQs are increasingly being incorporated into national and UN data collection instruments, including monitoring of the implementation of the CRPD and the Sustainable Development Goals, as well as being more widely used by researchers and advocates. However the use of the WGQs may not automatically translate into inclusive research practices—it should be recognised that people with more severe communication impairments may have difficulty understanding or responding to the WGQs themselves, without additional support. Robust data collection tools for data disaggregation are therefore not enough. Future research should implement data collection processes which are inclusive of PwCD so as to ensure that data pertaining to this population is accurate and reliable. In the context of the variability among PwCD, ensuring their inclusion may also require more complex solutions, and may have training and resource implications. Such resources may include advice from a speech and language therapist familiar with the local context and with strategies to support communication, and engagement with local organisations for persons with disabilities who specifically represent groups who may have communication needs (e.g. individuals with intellectual disability).

Accountability is the final principle of a HRBAD and it is of direct relevance here. Even inclusive research and data collection processes do not automatically translate into policy and practice–in some contexts people with disability may be over-researched but remain marginalised [88]. However the mandate to collect accurate and representative disability data remains important in order to monitor inequalities, with the next step then to ensure the data generated is used to meet the needs of people who may be marginalised [89]. Accountability in relation to data collection is complex and multifaceted. For the purposes of this paper we emphasise that data collection should be undertaken for a targeted purpose or goal and should inform policy and practice and should inform policy and practice, ultimately being answerable to the population who likely participated in the research with a sense that their contribution would inform policy and services relevant to their own life and wellbeing.

In this study, direct participation of PwCD in research on poverty was explored–a complex phenomenon which is usually assessed at the household level. Other domains, such as quality of life, experiences of, and access to, education, health and justice, are domains where direct participation may be particularly important.

### Key limitations and implications for future research

The main limitation for this critical review rest on the fact that analysis relied on what was *reported* about data collection tools and processes. Clearly it is possible, and even likely in some instances, that researchers or enumerators made adjustments ‘in the moment’. Informal visual analogue scales, or the use of supportive gesture may have been implemented but not reported. However, the vastly inconsistent prevalence findings, for example, indicate a need for transparency in reporting. The lack of detailed reporting on factors relating to method and study design warrant consideration. For transparency we suggest that issues of adjustment or communication support be clearly reported when used to allow for interpretation of the data.

Furthermore, the analysis was limited to consideration of communication disabilities as implicitly defined by the WGQs. The communication barriers experienced by people who are Deaf/deaf or hard of hearing were not explored in this study. The barriers to access intersect, but also have unique features and further exploration specific to the inclusion of Deaf / deaf and hard of hearing individuals in such research is warranted.

The use of *Poverty and Disability in Low- and Middle- Income Countries*: *A Systematic Review* [31] as a proxy for the systematic search of databases may also have led to some limitations. Firstly, the systematic review by Banks and colleagues, represents papers published up to March 2016, potentially excluding more recent relevant studies. However, our finding that the publication rate of studies including PwCD has not changed, suggests that this gap in literature is unlikely to significantly have impacted the findings or relevance of the critical review. Secondly, only comparison studies in English were included in the sample which may have resulted in the exclusion of relevant research in other languages. Furthermore, no qualitative studies were included and while the methods involved in qualitative data collection often includes interviews and focus groups (which would typically have high communication demands), the nature of qualitative research may also lend itself to increased opportunity for adaptation.

## Conclusion

People with communication disability may be subject to subtle and unrecognised restrictions in their right to freedom of expression and opinion [90, pg.1]. In this review, we have discussed the risk of disproportionate underrepresentation of PwCD in research on poverty. While rates of explicit exclusion were low, the extent of passive exclusion of PwCD from studies resulted in what arguably results in systematic underrepresentation. The limited volume of disaggregated data available for PwCD hints at a clear interaction between poverty and communication disability, and the reality for many people with more severe communication impairments is likely to be particularly daunting. In order to establish the true human cost of poverty and disability for PwCD, future research must improve the reliability and validity of data collection processes by using data collection tools which account for this population and administering data collection processes in a communicatively accessible way. A failure to provide adequate opportunity for participation of PwCD in research will, undoubtedly, leave those with communication disabilities behind in the pursuit of global poverty eradication.

## Supporting information

S1 File Table of papers included in critical review (alphabetical order). (DOCX)
