# Supplementary material for: Direct participation of people with communication disabilities in research on poverty and disabilities in low and middle income countries: A critical review
Source: PLoS One. 2021 Oct 14;16(10):e0258575. doi: 10.1371/journal.pone.0258575 (PMC8516265; doi:10.1371/journal.pone.0258575)
Supplement: S1 File — (DOCX) [file pone.0258575.s001.docx]

Supporting Information S1. Papers included in critical review (alphabetical order)

|  | Reference |
| --- | --- |
| 1 | Abas, M.A., and J.C. Broadhead. 1997. Depression and anxiety among women in an urban setting in Zimbabwe. *Psychological Medicine*, 27(1), 59-71. doi:10.1017/s0033291796004163 |
| 2 | Ali, N.S., S. Mahmud, A. Khan, and B.S. Ali. 2013. Impact of postpartum anxiety and depression on child's mental development from two peri-urban communities of Karachi, Pakistan: a quasi-experimental study. *Bmc Psychiatry*, 13, 274. doi:10.1186/1471-244X-13-274 |
| 3 | Anselmi, L., F.C. Barros, G.C. Minten, D.P. Gigante, B.L. Horta, and C.G. Victora. 2008. Prevalence and early determinants of common mental disorders in the 1982 birth cohort, Pelotas, Southern Brazil. *Revista de Saude Publica*, 42 Suppl 2(Suppl 2), 26-33.  doi:10.1590/s003489102008000900005 |
| 4 | Anselmi, L., A.M.B. Menezes, P.C. Hallal, F. Wehrmeister, H. Goncalves, F.C. Barros, J. Murray, and L.A. Rohde. 2012. Socioeconomic changes and adolescent psychopathology in a Brazilian birth cohort study. *Journal of Adolescent Health*, 51(Suppl 6), S5-S10.  doi:10.1016/j.jadohealth.2012.06.026 |
| 5 | Arguvanli, S., S. Akn, E.D. Safak, S. Mucuk, A. Ozturk, M.M. Mazcoglu, H.D. Kizilçay and S. Gocer. 2015. Prevalence of cognitive impairment and related risk factors in community-dwelling elderly in Kayseri, Turkey. *Turkish Journal of Medical Sciences*, 45(5), 1167-1172. doi:10.3906/sag-1406-149 |
| 6 | Arokiasamy, P., U. Uttamacharya, K. Jain, R.B. Biritwum, A.E. Yawson, F. Wu, Y. Guo, et al. 2015. The impact of multimorbidity on adult physical and mental health in low- and middle-income countries: what does the study on global ageing and adult health (SAGE) reveal? *BMC Medicine*, 13(1), 1-16. doi:10.1186/s12916-015-0402-8 |
| 7 | Ataguba, J.E., J. Akazili, and D. McIntyre. 2011. Socioeconomic-related health inequality in South Africa: Evidence from General Household Surveys. *International Journal for Equity in Health,* 10, 48. doi:10.1186/1475-9276-10-48 |
| 8 | Awas, M., D. Kebede, and A. Alem. 1999. Major mental disorders in Butajira, southern Ethiopia*. Acta Psychiatrica Scandinavica. Supplementum*, 397, 56-64. doi:10.1111/j.16000447.1999.tb10695.x |
| 9 | Ayazi, T., L. Lien, A. Eide, L. Swartz, and E. Hauff. 2014. Association between exposure to traumatic events and anxiety disorders in a post-conflict setting: a cross-sectional community study in South Sudan. *BMC Psychiatry*, 14(1), 1-22. doi:10.1186/1471-244X14-6. |
| 10 | Ball, H.A., S.H. Siribaddana, Y. Kovas, N. Glozier, P. McGuffin, A. Sumathipala, and M. Hotopf. 2010. Epidemiology and symptomatology of depression in Sri Lanka: a cross-sectional population-based survey in Colombo District. *Journal of Affective Disorders,* 123(1/3), 188196. doi:10.1016/j.jad.2009.08.014 |
| 11 | Basu, S., and A.C. King. 2013. Disability and Chronic Disease Among Older Adults in India: Detecting Vulnerable Populations Through the WHO SAGE Study. *American Journal of Epidemiology,* 178(11), 1620-1628. doi:10.1093/aje/kwt191 |
| 12 | Béria, J.U., B.C.W. Raymann, L.P. Gigante, A.C.L. Figueiredo, G. Jotz, R. Roithman, S. Selaimen da Costa, V. Garcez, C. Scherer, and Smith, A. 2007. Hearing impairment and socioeconomic factors: a population-based survey of an urban locality in southern Brazil. *Revista panamericana de salud publica [Pan American journal of public health],* 21(6), 381-387. doi:10.1590/S1020-49892007000500006 |
| 13 | Beydoun, M.A., and B.M. Popkin. 2005. The impact of socio-economic factors on functional status decline among community-dwelling older adults in China. *Social Science & Medicine (1982)*, 60(9), 2045-2057. doi:10.1016/j.socscimed.2004.08.063 |
| 14 | Blay, S.L., S.B. Andreoli, G.G. Fillenbaum, and F.L. Gastal. 2007. Depression morbidity in later life: prevalence and correlates in a developing country. *American Journal of Geriatric Psychiatry*, 15(9), 790-799. doi:10.1097.JGP.0b013e3180654179 |
| 15 | Blay, S.L., G.G. Fillenbaum, S.B. Andreoli, and F.L. Gastal. 2012. Prevalence and concomitants of arthritis in the elderly in Rio Grande do Sul, Brazil. *PLoS ONE,* 7(9), e45418. doi:10.1371/journal.pone.004541 |
| 16 | Blue, I. 2000. Individual and contextual effects on mental health status in Sao Paulo, Brazil. *Revista Brasileira de Psiquiatria,* 22(3), 116-123. doi:10.1590/s151644462000000300004. |
| 17 | Brown, R., S. Trapp, E. Berenz, T. Bigdeli, R. Acierno, T. Tran, L.T. Trung, et al. 2013. Pretyphoon socioeconomic status factors predict post-typhoon psychiatric symptoms in a Vietnamese sample. *Social Psychiatry & Psychiatric Epidemiology,* 48(11), 1721-1727. doi:10.1007/s00127-013-0684-0. |
| 18 | Chen, L., L. Wang, X.H. Qiu, X.X. Yang, Z.X. Qiao, Y.J. Yang, and Y. Liang. 2013. Depression among Chinese university students: prevalence and socio-demographic correlates. *PLoS One,* 8(3), e58379. doi:10.1371/journal.pone.0058379. |
| 19 | Chen, R., Z. Hu, L. Wei, Y. Ma, Z. Liu, and J.R. Copeland. 2011. Incident dementia in a defined older Chinese population. *PLoS ONE*, 6(9), e24817. doi:10.1371/ journal.pone.0024817 |
| 20 | Chen, R., Y. Ma, K. Wilson, Z. Hu, D. Sallah, J. Wang, L. Fan, R.-L. Chen, and J.R. Copeland. 2012. A multicentre community-based study of dementia cases and subcases in older people in China-the GMS-AGECAT prevalence and socio-economic correlates. *International Journal of Geriatric Psychiatry*, 27(7), 692-702. doi:10.1002/gps.2767. |
| 21 | Chen, R.L., L. Wei, Z. Hu, X. Qin, J.R. Copeland, and H. Hemingway. 2005. Depression in older people in rural China. *Archives of Internal Medicine,* 165(17), 2019-2025. doi:10.1001/archinte.165.17.2019. |
| 22 | Cockburn, N., D. Steven, K. Lecuona, F. Joubert, G. Rogers, C. Cook, and S. Polack. 2012. Prevalence, causes and socio-economic determinants of vision loss in Cape Town, South Africa. *PLoS ONE,* 7(2), e30718. doi:10.1371/journal.pone.0030718 |
| 23 | Coelho, F.M., R.T. Pinheiro, B.L. Horta, P.V. Magalhaes, C.M. Garcias, and C.V. Silva. 2009. Common mental disorders and chronic non-communicable diseases in adults: a population-based study. *Cadernos de saude publica,* 25(1), 59-67. doi:10.1590/s0102311x2009000100006 |
| 24 | Cordeiro de Andrade, K.R., M.T. Silva, T.F. Galvao, and M.G. Pereira. 2015. Functional disability of adults in Brazil: prevalence and associated factors. *Revista de Saude Publica,* 49, 89. doi:10.1590/S0034-8910.2015049005945 |
| 25 | Dandona, L., R. Dandona, S. Marmamula, G. Pyda, V. Kovai, M.N. Prasad, R.K. John, C.A. McCarthy, and G.N. Rao. 2001. Blindness in the Indian state of Andhra Pradesh. *Investigative Ophthalmology & Visual Science,* 42(5), 908-916. |
| 26 | Dandona, R., L. Dandona, M. Srinivas, P. Giridhar, M.N. Prasad, K. Vilas, C.A. McCarthy, and G.N. Rao. 2002. Moderate visual impairment in India: the Andhra Pradesh Eye Disease Study. *British Journal of Ophthalmology*, 86(4), 373-377. doi:10.1136/bjo.86.4.373 |
| 27 | Dang, H.M., B. Weiss, and L.T. Trung. 2016. Functional impairment and mental health functioning among Vietnamese children. *Social Psychiatry & Psychiatric Epidemiology,* 51(1), 39-47. doi:10.1007/s00127-015-1114-2 |
| 28 | Danquah, L., S. Polack, A. Brus, I. Mactaggart, C.P. Houdon, P. Senia, P. Gallien, and H. Kuper. 2015. Disability in post-earthquake Haiti: prevalence and inequality in access to services. *Disability and rehabilitation,* 37(12), 1082-1089.  doi: 10.1016/j.socscimed.2014.08.02110.3109/09638288.2014.956186. |
| 29 | Dasgupta, A., D. Ray, S. Roy, T. Sarkar, A. Ghosal, A. Das, and J. Pal. 2013. Depression among the Geriatric Population is a Matter of Concern: A Community Based Study in a Rural Area of West Bengal. *Nepal Journal of Epidemiology*, 3(4), 282-287. doi:10.3126/nje.v3i4.9515 |
| 30 | Emamian, M.H., H. Zeraati, R. Majdzadeh, M. Shariati, H. Hashemi, and A. Fotouhi. 2011. The gap of visual impairment between economic groups in Shahroud, Iran: a Blinder-Oaxaca decomposition. *American Journal of Epidemiology,* 173(12), 1463-1467. doi:10.1093/aje/kwr050 |
| 31 | Emamian, M.H., H. Zeraati, R. Majdzadeh, M. Shariati, H. Hashemi, E. Jafarzadehpur, and A. Fotouhi. 2013. Economic inequality in presenting near vision acuity in a middle-aged population: a Blinder-Oaxaca decomposition. *British Journal of Ophthalmology*, 97(9), 1100-1103. doi:10.1136/bjophthalmol-2013-30324 |
| 32 | Ergin, I., and A.E. Kunst. 2015. Regional inequalities in self-rated health and disability in younger and older generations in Turkey: the contribution of wealth and education. *BMC Public Health,* 15(987), 1-11. doi:10.1186/s12889-015-2273-5 |
| 33 | Escueta, M., K. Whetten, J. Ostermann, and K. O'Donnell. 2014. Adverse childhood experiences, psychosocial well-being and cognitive development among orphans and abandoned children in five low income countries. *BMC International Health & Human Rights,* 4(1), 113. doi:10.1186/1472-698X-14-6. |
| 34 | Falkingham, J.C., G. Chepngeno-Langat, C. Kyobutungi, A. Ezeh, and M. Evandrou. 2011. Does socioeconomic inequality in health persist among older people living in resource-poor urban slums? *Journal of Urban Health,* 88(Suppl 2), S381-400. doi:10.1007/s11524-0119559-4 |
| 35 | Fernandez-Nino, J.A., B.S. Manrique-Espinoza, I. Bojorquez-Chapela, and A. Salinas-Rodriguez. 2014. Income inequality, socioeconomic deprivation and depressive symptoms among older adults in Mexico. *PLoS One,* 9(9), e108127. doi:10.1371/journal.pone.0108127. |
| 36 | Fillenbaum, G.G., S.L. Blay, S.B. Andreoli, and F.L. Gastal. 2010. Prevalence and Correlates of Functional Status in an Older Community-Representative Sample in Brazil. *Journal of aging and health,* 22(3), 362-383. doi:10.1177/0898264309359307 |
| 37 | Filmer, D. 2008. Disability, poverty, and schooling in developing countries: Results from 14 household surveys. *World Bank Economic Review*, 22(1), 141-163. doi:10.1093/wber/lhm021 |
| 38 | Fortes, I., C. Paula, M. Oliveira, I. Bordin, J. de Jesus Mari, and L. Rohde. 2016. A cross-sectional study to assess the prevalence of DSM-5 specific learning disorders in representative school samples from the second to sixth grade in Brazil. *European Child & Adolescent Psychiatry,* 25(2), 195-207. doi:10.1007/s00787-015-0708-2. |
| 39 | Freeman. E.E., M.H. Roy-Gagnon, E. Samson, S. Haddad, M.J. Aubin, C. Vela, and M.V. Zunzunegui. 2013. The Global Burden of Visual Difficulty in Low, Middle, and High Income Countries. *PLoS ONE*, 8(5), e63315. doi:10.1371/journal.pone.0063315 |
| 40 | Gawde, N., M. Nasirabadi, N. Shah, and S. Nagaonkar. 2013. Psychiatric morbidity in an urban slum of Mumbai: cross sectional study. *Asian Journal of Psychiatry*, 6(6), 478-482. doi:10.1016/j.ajp.2013.05.005. |
| 41 | Graham, E., L.P. Jordan, and B.S. Yeoh. 2015. Parental migration and the mental health of those who stay behind to care for children in South-East Asia. *Social science & medicine (1982)*, 132, 225-235. doi: 10.1016/j.socscimed.2014.10.060. |
| 42 | Guerra, M., C.P. Ferri, A.L. Sosa, A. Salas, C. Gaona, V. Gonzales, G. Rojas de la Torre, and M. Prince. 2009. Late-life depression in Peru, Mexico and Venezuela: The 10/66 population-based study. *British Journal of Psychiatry,* 195(6), 510-515. doi:10.1192/bjp.bp.109.064055 |
| 43 | Guerra, R.O., B.E. Alvarado, and M.V. Zunzunegui. 2008. Life course, gender and ethnic inequalities in functional disability in a Brazilian urban elderly population. *Aging-Clinical & Experimental Research,* 20(1), 53-61. doi:10.1007/bf03324748 |
| 44 | Guo, S., D. Tian, X. Wang, Y. Xiao, H. He, Z. Qu, and X. Zhang. 2015. Protective Effects of Social Support Content and Support Source on Depression and Its Prevalence 6 Months after Wenchuan Earthquake. *Stress & Health: Journal of the International Society for the Investigation of Stress*, 31(5), 382-392. doi:10.1002/smi.2563. |
| 45 | Gureje, O., L. Kola, and E. Afolabi. 2007. Epidemiology of major depressive disorder in elderly Nigerians in the Ibadan Study of Ageing: a community-based survey. *Lancet,* 370(9591), 957-964. doi:10.1016/s0140-6736(07)61446-9. |
| 46 | Gureje, O., A. Ogunniyi, L. Kola, and E. Afolabi. 2006. Functional disability in elderly Nigerians: Results from the Ibadan Study of Aging. *Journal of the American Geriatrics Society,* 54(11), 1784-1789. doi: 10.1111/j.1532-5415.2006.00944.x |
| 47 | Habtamu, E., T. Wondie, S. Aweke, Z. Tadesse, M. Zerihun, Z. Zewdie, K. Callahan, et al. 2015. Trachoma and Relative Poverty: A Case-Control Study. *PLoS Neglected Tropical Diseases,* 9(11), e0004228. doi:10.1371/journal. pntd.0004228 |
| 48 | Halpern, R., A.J.D. Barros, A. Matijasevich, I.S. Santos, C.G. Victora, and F.C. Barros. 2008. Developmental status at age 12 months according to birth weight and family income: A comparison of two Brazilian birth cohorts. *Cadernos de Saude Publica,* 24(SUPPL.3), S444S450. doi:10.1590/s0102-311x2008001500010 |
| 49 | Hanandita, W. and G. Tampubolon. 2014. Does poverty reduce mental health? An instrumental variable analysis. *Social science & medicine (1982)*, 113, 59-67. doi:10.1016/j.socscimed.2014.05.005. |
| 50 | Herrera, E., P. Caramelli, A.S.B. Silveira, and R. Nitrini. 2002. Epidemiologic survey of dementia in a community-dwelling Brazilian population. *Alzheimer Disease & Associated Disorders,* 16(2), 103-108. doi:10.1097/01.WAD.0000020202.50697.df. |
| 51 | Ho, V.H. and I.R. Schwab. 2001. Social economic development in the prevention of global blindness. *British Journal of Ophthalmology,* 85(6), 653-657. doi:10.1136/bjo.85.6.653 |
| 52 | Hoogeveen, J.G. 2005. Measuring welfare for small but vulnerable groups: Poverty and disability in Uganda. *Journal of African Economies*, 14(4), 603-631. doi:10.1093/jae/eji020 |
| 53 | Hosseinpoor, A.R., N. Bergen, S. Mendis, S. Harper, E. Verdes, A. Kunst, and S. Chattterji. 2012. Socioeconomic inequality in the prevalence of noncommunicable diseases in low- and middle-income countries: results from the World Health Survey. *BMC Public Health*, 12, 474. doi:10.1186/1471-2458-12-474 |
| 54 | Hosseinpoor, A.R., J.A.S. Williams, J. Gautam, A. Posarac, A. Officer, E. Verdes, N. Kostanjse, and S. Chatterji. 2013. Socioeconomic inequality in disability among adults: a multicountry study using the World Health Survey. *American Journal of Public Health,* 103(7), 1278-1286. doi:10.2105/AJPH.2012.301115 |
| 55 | Husain, N., C. Nasim, J. Farhat, B. Tomenson, S. Ishaq, M. Ilyas, and I.B. Chaudhry. 2014. Prevalence and risk factors for psychological distress and functional disability in urban Pakistan. *WHO South East Asia Journal of Public Health*, 3(2), 144-153. doi:10.4103/22243151.206730 |
| 56 | Ibrahim, A.K., S.J. Kelly, and C. Glazebrook. 2012. Analysis of an Egyptian study on the socioeconomic distribution of depressive symptoms among undergraduates. *Social psychiatry and psychiatric epidemiology*, 47(6), 927-937. doi:10.1007/s00127-011-0400x. |
| 57 | Islam, M., M. Ali, P. Ferroni, P. Underwood, and M. FaruqAlam. 2003. Prevalence of psychiatric disorders in an urban community in Bangladesh. *General hospital psychiatry,* 25(5), 353357. doi: 10.1016/s0163-8343(03)00067-7 |
| 58 | Jenkins, R., C. Othieno, L. Ongeri, P. Sifuna, M. Ongecha, J. Kingora, D. Kiima, R. Omollo, and B. Ogutu. 2015. Common mental disorder in Nyanza province, Kenya in 2013 and its associated risk factors -an assessment of change since 2004, using a repeat household survey in a demographic surveillance site. *BMC Psychiatry,* 15, 1-12. doi:10.1186/s12888-015-0693-5. |
| 59 | Jiang, N.M., F. Tofail, R.J. Scharf, S.N. Moonah, M. Taniuchi, J.Z. Ma, J.D. Hamadani, et al. 2014. Febrile illness and pro-inflammatory cytokines are associated with lower neurodevelopmental scores in Bangladeshi infants living in poverty. *BMC Paediatrics,* 14(1), 50. doi:10.1186/1471-2431-14-50 |
| 60 | Kawakami, N., E.A. Abdulghani, J. Alonso, E.J. Bromet, R. Bruffaerts, J.M. Caldas-de-almeida, W.T. Chiu, et al. 2012. Early-Life Mental Disorders and Adult Household Income in the World Mental Health Surveys. *Biological psychiatry (1969),* 72(3), 228-237. doi:10.1016/j.biopsych.2012.03.009 |
| 61 | Kawakatsu, Y., S. Kaneko, and M. Karama. 2012. Honda S. Prevalence and risk factors of neurological impairment among children aged 6-9 years: from population based cross sectional study in western Kenya. *BMC Paediatrics,* 12, 186. doi:10.1186/1471-2431-12186 |
| 62 | Keskinoglu, P., H. Giray, M. Pıcakcıefe, N. Bilgic, and R. Ucku. 2006. The prevalence and risk factors of dementia in the elderly population in a low socio-economic region of Izmir, Turkey. *Archives of Gerontology & Geriatrics,* 43(1), 93-100. doi:10.1016/j.archger.2005.09.006. |
| 63 | Kilzieh, N., S. Rastam, K.D. Ward, and W. Maziak. 2010. Gender, depression and physical impairment: an epidemiologic perspective from Aleppo, Syria. *Social Psychiatry & Psychiatric Epidemiology*, 45(6), 595-602. doi:10.1007/s00127-009-0076-7 |
| 64 | Kulkarni, R.S. and R.L. Shinde. 2014. Depression and Its Associated Factors in Older Indians: A Study Based on Study of Global Aging and Adult Health (SAGE)-2007. *Journal of aging and health,* 27(4), 622-649. doi:10.1177/0898264314556617. |
| 65 | Kumar, R., A.K. Aggarwal, M. Kaur, and S.D. Lyengar. 1997. Factors influencing psychosocial development of preschool children in a rural area of Haryana, India. *Journal of Tropical Paediatrics,* 43(6), 324-329. doi:10.1093/tropej/43.6.324. |
| 66 | Kumar, R., B. Anupama, B. Roli, and G.G. Agarwal. 2013. Prevalence and risk factors for neurological disorders in children aged 6 months to 2 years in northern India. *Developmental Medicine & Child Neurology*, 55(4), 348-356. doi:10.1111/dmcn.12079 |
| 67 | Kuper, H., A. Monteath-van Dok, K. Wing, L. Danquah, J. Evans, M. Zuurmond, and J. Gallenitti. 2014. The impact of disability on the lives of children; cross-sectional data including 8,900 children with disabilities and 898,834 children without disabilities across 30 countries. *PLoS One*, 9(9), e107300. doi:10.1371/journal.pone.0107300. |
| 68 | Kuper, H., V. Nyapera, J. Evans, D. Munyendo, M. Zuurmond, S. Frison, V. Mwenda, D. Otieno, and J. Kisia. 2015. Malnutrition and childhood disability in Turkana, Kenya: Results from a case-control study. *PloS one,* 10(12), e0144926. doi:10.1371/journal.pone.0144926 |
| 69 | Kuper, H., S. Polack, A. Foster, C. Eusebio, W. Mathenge, and Z. Wadud. 2008. A case-control study to assess the relationship between poverty and visual impairment from cataract in Kenya, the Philippines, and Bangladesh. *PLoS Medicine,* 5(12), 1716-1728. doi:10.1371/journal.pmed.0050244 |
| 70 | Lei, X., X. Sun, J. Strauss, P. Zhang, and Y. Zhao. 2014. Depressive symptoms and SES among the mid-aged and elderly in China: Evidence from the China Health and Retirement Longitudinal Study national baseline. *Social Science & Medicine,* 120, 224-232. doi:10.1016/j.socscimed.2014.09.028. |
| 71 | Levinson, D., M.D. Lakoma, M. Petukhova, M. Schoenbaum, A.M. Zaslavsky, M. Angermeyer, G. Borges, et al. 2010. Associations of serious mental illness with earnings: results from the WHO World Mental Health surveys. *British journal of psychiatry,* 197(AOU), 114-121. doi:10.1192/bjp.bp.109.073635 |
| 72 | Li, N., W. Du, L. Zhang, G. Chen, and X. Zheng. 2015a. Prevalence and functions of mental disability caused by mood disorders in China: A national sample. *Journal of affective disorders,* 180, 10-3. doi:10.1016/j.jad.2015.03.016. |
| 73 | Li, N., L. Pang, G. Chen, X. Song, J. Zhang, and X. Zheng. 2011. Risk factors for depression in older adults in Beijing. *Canadian journal of psychiatry [Revue canadienne de psychiatrie]*, 56(8), 466-473. doi:10.1177/070674371105600804 |
| 74 | Li, N., L. Pang, W. Du, G. Chen, and X. Zheng. 2012. Association between poverty and psychiatric disability among Chinese population aged 15–64 years. *Psychiatry Research,* 200(2/3), 917-920. doi:10.1016/j.psychres.2012.05.026. |
| 75 | Li, N., L. Zhang, W. Du, L. Pang, C. Guo, G. Chen, and X. Zheng. 2015b. Prevalence of dementiaassociated disability among chinese older adults: Results from a national sample survey. *American Journal of Geriatric Psychiatry,* 23(3), 320-325. doi:10.1016/j.jagp.2014.06.002 |
| 76 | Lima, M.S., J.U. Beria, E. Tomasi, A.T. Conceicao, and J.J. Mari. 1996. Stressful life events and minor psychiatric disorders: an estimate of the population attributable fraction in a Brazilian community-based study. *The International Journal of Psychiatry in Medicine*, 26(2), 211-222. doi:10.2190/W4U4-TCTX-164J-KMAB |
| 77 | Lin, T., N. Li, W. Du, X. Song, and X. Zheng. 2013. Road traffic disability in China: prevalence and socio-demographic disparities. *Journal of public health,* 35(4), 541-547. doi:10.1093/pubmed/fdt003. |
| 78 | Liu, J., I. Chi, G. Chen, X. Song, and X. Zheng. 2009. Prevalence and correlates of functional disability in Chinese older adults. *Geriatrics & gerontology international,* 9(3), 253-261. doi:10.1111/j.1447-0594.2009.00529.x |
| 79 | Liu, J., F. Yan, X. Ma, H.L. Guo, Y.L. Tang, J.J. Rakofsky, X-M. Wu, et al. 2015a. Prevalence of major depressive disorder and socio-demographic correlates: Results of a representative household epidemiological survey in Beijing, China. *Journal of affective disorders,* 179, 7481. doi:10.1016/j.jad.2015.03.009. |
| 80 | Liu, T., L. Zhang, L. Pang, N. Li, G. Chen, and X. Zheng. 2015b. Schizophrenia-related disability in China: prevalence, gender, and geographic location. *Psychiatric services,* 66(3), 249-257. doi:10.1176/appi.ps.201400032. |
| 81 | Lopes, M.A., S.R. Hototian, S.E.Z. Bustamante, D. Azevedo, M. Tatsch, M.C. Bazzarella, J. Litvoc, and C.M.C. Bottino. 2007. Prevalence of cognitive and functional impairment in a community sample in Ribeirão Preto, Brazil. *International Journal of Geriatric Psychiatry,* 22(8), 770776. doi:10.1002/gps.1737. |
| 82 | Loyalka, P., L. Liu, G. Chen, and X. Zheng. 2014. The cost of disability in China. *Demography,* 51(1), 97-118. doi:10.1007/s13524-013-0272-7. |
| 83 | Ludermir, A.B. and G. Lewis. 2001. Links between social class and common mental disorders in Northeast Brazil. *Social psychiatry and psychiatric epidemiology,* 36(3), 101-107. doi:10.1007/s001270050297 |
| 84 | Ma, X., Y.T. Xiang, Z.J. Cai, J.Y. Lu, S.R. Li, Y.Q. Xiang, Y-Z. Hou, et al. 2009. Generalized Anxiety Disorder in China: Prevalence, Sociodemographic Correlates, Comorbidity, and Suicide Attempts. *Perspectives in Psychiatric Care,* 45(2), 119-127. doi:10.1111/j.17446163.2009.00212.x. |
| 85 | Malhotra, R., A. Chan, and T. Ostbye. 2010. Prevalence and correlates of clinically significant depressive symptoms among elderly people in Sri Lanka: findings from a national survey. *International Psychogeriatrics,* 22(2), 227-236. doi:10.1017/S1041610209990871 |
| 86 | Marella, M., N.L. Huq, A. Devine, S.M. Baker, M.A. Quaiyum, and J.E. Keeffe. 2015. Prevalence and correlates of disability in Bogra district of Bangladesh using the rapid assessment of disability survey. *BMC Public Health,* 15(867), 1-9. doi:10.1186/s12889-015-2202-7 |
| 87 | Medina-Mora, M.E., G. Borges, C. Lara, C. Benjet, J. Blanco, C. Fleiz, J. Villatorro, E. Rojas, et al. 2005. Prevalence, service use, and demographic correlates of 12-month DSM-IV psychiatric disorders in Mexico: results from the Mexican National Comorbidity Survey. *Psychological medicine,* 35(12), 1773-1783. doi:10.1017/s0033291705005672. |
| 88 | Melzer, D. and M.I. Parahyba. 2004. Socio-demographic correlates of mobility disability in older Brazilians: results of the first national survey. *Age & Ageing,* 33(3), 253-259. DOI: 10.1093/ageing/afh075 |
| 89 | Minh, H.V., K.B. Giang, N.T. Liem, M. Palmer, N.P. Thao, and L.B. Duong. 2015. Estimating the extra cost of living with disability in Vietnam. *Global Public Health,* 10, S70-S79. doi:10.1080/17441692.2014.971332. |
| 90 | Minicuci, N., R.B. Biritwum, G. Mensah, A.E. Yawson, N. Naidoo, S. Chatterji, and P. Kowal. 2014. Sociodemographic and socioeconomic patterns of chronic non-communicable disease among the older adult population in Ghana. *Global health action,* 7(1), 21292. doi:10.3402/gha.v7.21292. |
| 91 | Mitra. S., A. Posarac, and B. Vick. 2013. Disability and Poverty in Developing Countries: A Multidimensional Study. *World Development,* 41, 1-18. doi:10.1016/j.worlddev.2012.05.024. |
| 92 | Mokhtari, M., S.F. Dehghan, M. Asghari, U. Ghasembaklo, G. Mohamadyari, S.A. Azadmanesh, and E. Akbari. 2013. Epidemiology of mental health problems in female students: a questionnaire survey. *Journal of epidemiology and global health,* 3(2), 83-88. doi:10.1016/j.jegh.2013.02.005.S |
| 93 | Mont, D. and N.V. Cuong. 2011. Disability and poverty in Vietnam. *The World Bank Economic Review,* 25(2), 323-359. doi:10.1093/wber/lhr019 |
| 94 | Myer, L., D.J. Stein, A. Grimsrud, S. Seedat, and D.R. Williams. 2008. Social determinants of psychological distress in a nationally-representative sample of South African adults. *Social science & medicine (1982)*, 66(8), 1828-1840. doi:10.1016/j.socscimed.2008.01.025. |
| 95 | Nakua, E.K., E. Otupiri, V.M. Dzomeku, E. Owusu-Dabo, P. Agyei-Baffour, A.E. Yawson, G. Folson, and S. Hewlett. 2015. Gender disparities of chronic musculoskeletal disorder burden in the elderly Ghanaian population: study on global ageing and adult health (SAGE WAVE 1). *BMC Musculoskeletal Disorders,* 16(204), 1-10. doi:10.1186/s12891-015-0666-3 |
| 96 | Natale, J.E., J.G. Joseph, R. Bergen, R.D. Thulasiraj, and L. Rahmathullah. 1992. Prevalence of childhood disability in a southern indian city - independent effect of small differences in social-status. *International Journal of Epidemiology,* 21(2), 367-372. doi:10.1093/ije/21.2.367. |
| 97 | Nguyen, A.J., E.E. Haroz, T. Mendelson, and J. Bass. 2016. Symptom Endorsement and Sociodemographic Correlates of Postnatal Distress in Three Low Income Countries. *Depression Research & Treatment,* 1-11. doi:10.1155/2016/1823836. |
| 98 | Nguyen, T.T., T.D. Tran, T. Tran, B. La, H. Nguyen, and J. Fisher. 2015. Postpartum change in common mental disorders among rural Vietnamese women: incidence, recovery and risk and protective factors. *The British journal of psychiatry: the journal of mental science,* 206(2), 110-115. doi:10.1192/bjp.bp.114.149138. |
| 99 | Norris, F.H., A.D. Murphy, C.K. Baker, J.L. Perilla, F.G. Rodriguez, and J.J. Rodriguez. 2003. Epidemiology of trauma and posttraumatic stress disorder in Mexico. *Journal of abnormal psychology,* 112(4), 646-656. Epub 2003/12/17. doi:10.1037/0021-843x.112.4.646. |
| 100 | Ou, J.J., L.J. Shi, G.L. Xun, C. Chen, R.R. Wu, X.R. Luo, F-Y. Zhang, and J.P. Zhao. 2015. Employment and financial burden of families with preschool children diagnosed with autism spectrum disorders in urban China: results from a descriptive study. *BMC Psychiatry,* 15(3), 1-8. doi:10.1186/s12888-015-0382-4. |
| 101 | Palmer, M.G. 2014. Inequalities in universal health coverage: evidence from Vietnam. *World Development,* 64, 384-394. doi:10.1016/j.worlddev.2014.06.008 |
| 102 | Palmer, M.G., T. Nguyen Thi Minh, Q. Quach Thi Ngoc, D. Dang Sy, H. Hoang Van, and H.L. Berry. 2012. Disability measures as an indicator of poverty: A case study from Vietnam. *Journal of International Development,* 24, S53-S68. doi:10.1002/jid.1715 |
| 103 | Patel, V., B.R. Kirkwood, S. Pednekar, H. Weiss, and D. Mabey. 2006. Risk factors for common mental disorders in women - Population-based longitudinal study. *British Journal of Psychiatry,* 189, 547-555. doi:10.1192/bjp.bp.106.022558. |
| 104 | Peres, M.A., J.L. Bastos, R.G. Watt, A.J. Xavier, P.R. Barbato, and E. D'Orsi. 2015. Tooth loss is associated with severe cognitive impairment among older people: findings from a population-based study in Brazil. *Aging & Mental Health,* 19(10), 876-884. doi:10.1080/13607863.2014.977770. |
| 105 | Petresco, S., L. Anselmi, I. Santos, A. Barros, B. Fleitlich-Bilyk, F. Barros, and A. Matijasevich. 2014. Prevalence and comorbidity of psychiatric disorders among 6-year-old children: 2004 Pelotas Birth Cohort. *Social Psychiatry & Psychiatric Epidemiology,* 49(6), 975-983. doi:10.1007/s00127-014-0826-z. |
| 106 | Pham, H.L., M. Kizuki, T. Takano, K. Seino, and M. Watanabe. 2013. Out-of-pocket Costs of Disabilities and Their Association with Household Socioeconomic Status Among Schoolaged Children in Vietnam. *Journal of Rural Medicine,* 8(2), 212-221. doi:10.2185/jrm.287 |
| 107 | Pheula, G.F., L.A. Rohde, and M. Schmitz. 2011. Are family variables associated with ADHD, inattentive type? A case―control study in schools. *European child & adolescent psychiatry,* 20(3), 137-145. doi:10.1007/s00787-011-0158-4 |
| 108 | Ploubidis, G.B., W. Mathenge, B. Stavola, E. Grundy, A. Foster, and H. Kuper. 2013. Socioeconomic position and later life prevalence of hypertension, diabetes and visual impairment in Nakuru, Kenya. *International Journal of Public Health,* 58(1), 133-141. doi:10.1007/s00038-012-0389-2 |
| 109 | Quadros, LdCMd., LdA. Quevedo, JVdS. Motta, A. Carraro, F.G. Ribeiro, B.L. Horta, and D.P. Gigante. 2015. Social Mobility and Mental Disorders at 30 Years of Age in Participants of the 1982 Cohort, Pelotas, Rio Grande Do Sul – RS. *PLoS ONE,* 10(10), 1-11. doi:10.1371/journal.pone.0136886. |
| 110 | Rajkumar, A.P., P. Thangodurai, P. Senthilkumar, K. Goyathri, M. Prince, and K.S. Jacob. 2009. Nature, prevalence and factors associated with depression among the elderly in a rural south Indian community. *International Psychogeriatrics,* 21(2), 372-378. doi:10.1017/s1041610209008527. |
| 111 | Razzaque, A., L. Nahar, M.A. Khanam, and P.K. Streatfield. 2010. Socio-demographic differentials of adult health indicators in Matlab, Bangladesh: self-rated health, health state, quality of life and disability level. *Global Health Action,* 3, 70-77. doi:10.3402/gha.v3i0.4618. |
| 112 | Rocha, S.V., M.M. de Almeida, T.M. de Araujo, and J.S.Jr. Virtuoso. 2010. Prevalence of common mental disorders among the residents of urban areas in Feira de Santana, Bahia. *Revista brasileira de epidemiologia [Brazilian journal of epidemiology],* 13(4), 630-640. doi:10.1590/s1415-790x2010000400008 |
| 113 | Saha, S.K., D. Sanyal, A. Bhattacharyya, R. Bhattacharyya, N. Barman, and A. Mukherjee. 2010. A study on cognitive status of 50 years and above aged non-demented women in a rural area of West Bengal. *Journal of the Indian Medical Association,* 108(11), 726-729. |
| 114 | Santos, I.S., A. Matijasevich, A.J.D. Barros, and F.C. Barros. 2014. Antenatal and postnatal maternal mood symptoms and psychiatric disorders in pre-school children from the 2004 Pelotas Birth Cohort. *Journal of Affective Disorders,* 164, 112-117. doi:0.1016/j.jad.2014.04.033. |
| 115 | Scazufca, M., P.R. Menezes, R. Araya, VDd. Rienzo, O.P. Almeida, D. Gunnell, and D.A. Lawlor. 2008. Risk factors across the life course and dementia in a Brazilian population: results from the Sao Paulo Ageing & Health Study (SPAH). *International Journal of Epidemiology,* 37(4), 879-890. doi:10.1093/ije/dyn125 |
| 116 | Sengupta, P. and A.I. Benjamin. 2015. Prevalence of depression and associated risk factors among the elderly in urban and rural field practice areas of a tertiary care institution in Ludhiana. *Indian Journal of Public Health,* 59(1), 3-8. doi:10.4103/0019-557X.152845 |
| 117 | Sengupta, P., A.I. Benjamin, S. Yashpal, and G. Ashoo. 2014. Prevalence and correlates of cognitive impairment in a north Indian elderly population. *WHO South East Asia Journal of Public Health,* 3(2), 135-143. doi:10.4103/2224-3151.206729 |
| 118 | Shams, G., E. Foroughi, Y. Esmaili, H. Amini, and N. Ebrahimkhani. 2011. Prevalence rates of obsessive-compulsive symptoms and psychiatric comorbidity among adolescents in Iran. *Acta Medica Iranica,* 49(10), 680-687. |
| 119 | Sharifi, V., M. Amin-Esmaeili, A. Hajebi, A. Motevalian, R. Radgoodarzi, M. Hefazi, and A. Rahimi-Movagha. 2015. Twelve-month prevalence and correlates of psychiatric disorders in Iran: the Iranian Mental Health Survey, 2011. *Archives of Iranian medicine,* 18(2), 76-84. doi:015182/aim.004. |
| 120 | Soares, W.B., S.R.I Ribeiz, D. Bassitt, M.C. de Oliveira, and C.M.C Bottino. 2015. Psychotic symptoms in older people without dementia from a brazilian community-based sample. *International Journal of Geriatric Psychiatry,* 30, 437-445. doi:10.1002/gps.415 |
| 121 | Sosa, A.L., E. Albanese, B.C.M. Stephan, M. Dewey, D. Acosta, C.P. Ferri, M. Guerra, et al. 2012. Prevalence, Distribution, and Impact of Mild Cognitive Impairment in Latin America, China, and India: A 10/66 Population-Based Study. *Plos Medicine*, 9(2), 1-11. doi:10.1371/journal.pmed.1001170. |
| 122 | Sozmen, K. and B. Unal. 2014. Socioeconomic Inequalities in non-communicable diseases and self assessed health in Turkey. *Iranian Journal of Public Health*, 43(6), 736-748. |
| 123 | Subbaraman, R., L. Nolan, T. Shitole, S. Kiran, S. Shrutika, K. Sood, M. Nanarkar, et al. 2014. The psychological toll of slum living in Mumbai, India: a mixed methods study. *Social Science & Medicine,* 119, 155-169. doi:10.1016/j.socscimed.2014.08.021 |
| 124 | Taha, A.A., S.R. Pratt, T.M. Farahat, G.M. Abdel-Rasoul, M.A. Albtanony, A-L.E. Elrashiedy, H.R. Alwakeel, and A. Zein. 2010. Prevalence and Risk Factors of Hearing Impairment Among Primary-School Children in Shebin El-Kom District, Egypt. *American Journal of Audiology,* 19(1), 46-60. doi:10.1044/1059-0889(2010/09-0030. |
| 125 | Topuzoglu, A., T. Binbay, H. Ulas, H. Elbi, F.A. Tanik, N. Zagli, and K. Alptekin. 2015. The epidemiology of major depressive disorder and subthreshold depression in Izmir, Turkey: Prevalence, socioeconomic differences, impairment and help-seeking. *Journal of Affective Disorders,* 181, 78-86. doi:10.1016/j.jad.2015.04.017. |
| 126 | Trani, J-F., P. Bakhshi, J. Kuhlberg, S.S. Narayanan, H. Venkataraman, N.N. Mishra, N.E. Groce, S. Jadhav, and S. Deshpande. 2015a. Mental illness, poverty and stigma in India: a case-control study. *BMJ open,* 5(2), e006355. doi:10.1136/bmjopen-2014-006355. |
| 127 | Trani, J-F., P. Bakhshi, S. Myers Tlapek, D. Lopez, and F. Gall. 2015b. Disability and Poverty in Morocco and Tunisia: A Multidimensional Approach. *Journal of Human Development & Capabilities,* 16(4), 518-548. doi:10.1080/19452829.2015.1091808. |
| 128 | Trani, J-F., M. Biggeri and V. Mauro. 2013. The Multidimensionality of Child Poverty: Evidence from Afghanistan. *Social Indicators Research,* 112(2), 391-416. doi:10.1007/s11205-0130254-6 |
| 129 | Trani, J-F. and M. Loeb. 2012. Poverty and disability: A vicious circle? Evidence from Afghanistan and Zambia. *Journal of International Development*, 24, S19-S52. doi:10.1002/jid.1709. |
| 130 | Vukovic, D., V. Bjegovic, and G. Vukovic. 2008. Prevalence of chronic diseases according to socioeconomic status measured by wealth index: health survey in Serbia. *Croatian Medical Journal*, 49(6), 832-841. doi:10.3325/cmj.2008.49.83 |
| 131 | Wandera, S.O., J. Ntozi, and B. Kwagala, B. 2014. Prevalence and correlates of disability among older Ugandans: evidence from the Uganda National Household Survey. *Global Health Action,* 7, 25686. doi:10.3402/gha.v7.25686 |
| 132 | Wang, H., Y.Y. Xiaozhao, Y. Tingzhong, R.R. Cottrell, Y. Lingwei, X. Feng, and S. Jiang. 2015a. Socioeconomic inequalities and mental stress in individual and regional level: a twenty one cities study in China. *International Journal for Equity in Health,* 14(1), 1-7. doi:10.1186/s12939-015-0152-4. |
| 133 | Wang, L., Z. Feng, G. Yang, Y. Yang, Q. Dai, C. Hu, K. Liub, Y. Guan, et al. 2015b. The epidemiological characteristics of depressive symptoms in the left-behind children and adolescents of Chongqing in China. *Journal of Affective Disorders,* 177, 36-41. doi:10.1016/j.jad.2015.01.002 |
| 134 | Wang. S., C. Kou, Y. Liu, B. Li, Y. Tao, C. D'Arcy, J. Shi, et al. 2015c. Rural-urban differences in the prevalence of chronic disease in northeast China. *Asia-Pacific journal of public health / Asia-Pacific Academic Consortium for Public Health,* 27(4), 394-406. doi:10.1177/1010539514551200. |
| 135 | Wang, Z.J., W. Du, L. Pang, L. Zhang, G. Chen, and X. Zheng. 2015d. Wealth inequality and mental disability among the Chinese population: a population based study. *International Journal of Environmental Research and Public Health,* 12(10), 13104-13117. doi:10.3390/ijerph121013104 |
| 136 | Weobong, B., S. Soremekun, A.H. Ten Asbroek, S. Amenga-Etego, S. Danso, S. Owusu-Agyei, M. Prince, and B.R. Kirkwood. 2014. Prevalence and determinants of antenatal depression among pregnant women in a predominantly rural population in Ghana: The DON populationbased study (English). *Journal of Affective Disorders,* 165, 1-7. doi:10.1016/j.jad.2014.04.009 |
| 137 | Williams, J.S., N. Ng, K. Peltzer, A. Yawson, R. Biritwum, T. Maximova, F. Wu, P. Arokiasamy, P. Kowal, and S. Chatterji. 2015. Risk factors and disability associated with low back pain in older adults in low- and middleincome countries. Results from the WHO study on global AGEing and adult health (SAGE). *PLoS ONE,* 10(6), e0127880. doi:10.1371/journal.pone.0127880 |
| 138 | Wu, F., Y. Guo, P. Kowal, Y. Jiang, M. Yu, X. Li, Y. Zheng, and J. Xu. 2013. Prevalence of Major Chronic Conditions among Older Chinese Adults: The Study on Global AGEing and Adult Health (SAGE) Wave 1. *PLoS ONE,* 8(9), e74176. doi:10.1371/journal.pone.0074176 |
| 139 | Wu, Z., J. Xu, and L. He. 2014. Psychological consequences and associated risk factors among adult survivors of the 2008 Wenchuan earthquake. *BMC Psychiatry,* 14, 126. doi:10.1186/1471-244x-14-126. |
| 140 | Xavier Gomez-Olive, F., M. Thorogood, B.D. Clark, K. Kahn, and S.M. Tollman. 2010. Assessing health and well-being among older people in rural South Africa. *Global Health Action,* 3(Suppl 2), 23-35. doi:10.3402/gha.v3i0.2126 |
| 141 | Xiang, Y.T., X. Ma, Z.J. Cai, S.R. Li, Y.Q. Xiang, H.L. Guo, Y.-Z. Hou, et al. 2008. Prevalence and socio-demographic correlates of schizophrenia in Beijing, China. *Schizophrenia research,* 102(1-3), 270-277. doi:10.1016/j.schres.2008.04.009 |
| 142 | Xie, Z.H., S.Y. Bo, X.T. Zhang, M. Liu, Z.X. Zhang, X.L. Yang, S.-R. Ji, et al. 2008. Sampling survey on intellectual disability in 0 similar to 6-year-old children in China. *Journal of Intellectual Disability Research*, 52, 1029-1038. doi:10.1111/j.1365-2788.2008.01048.x. |
| 143 | Zainal, M., L. Masran, and A.R. Ropilah. 1998. Blindness and visual impairment amongst rural Malays in Kuala Selangor, Selangor. *Medical* *Journal of Malaysia,* 53(1), 46-50. |
| 144 | Zheng, X., R. Chen, N. Li, W. Du, L. Pei, J. Zhang, Y. Ji, X. Song, L. Tan, and R. Yang. 2012. Socioeconomic status and children with intellectual disability in China. *Journal of Intellectual Disability Research,* 56(2), 212-220. doi:10.1111/j.1365-2788.2011.01470.x |
| 145 | Zhou, X., B. Bi, L. Zheng, Z. Li, H. Yang, H. Song, and Y. Sun. 2014. The prevalence and risk factors for depression symptoms in a rural Chinese sample population. *PLoS ONE,* 9(6), 1-9. doi:10.1371/journal.pone.0099692 |
